# A bacterial secreted translocator hijacks riboregulators to control type III secretion in response to host cell contact

**DOI:** 10.1371/journal.ppat.1007813

**Published:** 2019-06-07

**Authors:** Maria Kusmierek, Jörn Hoßmann, Rebekka Witte, Wiebke Opitz, Ines Vollmer, Marcel Volk, Ann Kathrin Heroven, Hans Wolf-Watz, Petra Dersch

**Affiliations:** 1 Department of Molecular Infection Biology, Helmholtz Centre for Infection Research, Braunschweig, Germany; 2 Institute for Infectiology, University of Münster, Germany; 3 Department of Molecular Biology, Umea University, Sweden; Tufts University, UNITED STATES

## Abstract

Numerous Gram-negative pathogens use a Type III Secretion System (T3SS) to promote virulence by injecting effector proteins into targeted host cells, which subvert host cell processes. Expression of T3SS and the effectors is triggered upon host cell contact, but the underlying mechanism is poorly understood. Here, we report a novel strategy of *Yersinia pseudotuberculosis* in which this pathogen uses a secreted T3SS translocator protein (YopD) to control global RNA regulators. Secretion of the YopD translocator upon host cell contact increases the ratio of post-transcriptional regulator CsrA to its antagonistic small RNAs CsrB and CsrC and reduces the degradosome components PNPase and RNase E levels. This substantially elevates the amount of the common transcriptional activator (LcrF) of T3SS/Yop effector genes and triggers the synthesis of associated virulence-relevant traits. The observed hijacking of global riboregulators allows the pathogen to coordinate virulence factor expression and also readjusts its physiological response upon host cell contact.

## Author summary

Many bacterial pathogens sense contact to host cells and respond by inducing expression of crucial virulence factors. This includes the type III secretion systems (T3SSs) and their substrates, which manipulate different host cell functions to promote colonization and survival of the pathogen within its host. In this study, we used enteropathogenic *Yersinia pseudotuberculosis* to elucidate the molecular mechanism of how cell contact is transmitted and translated to trigger this process. We found that multiple global riboregulators control the decay and/or translation of the major transcriptional activator of the T3SS. In the absence of cell contact, these important RNA regulators are coopted by one of the substrate proteins of the T3SS to repress expression of the secretion machinery. Translocation of the substrate protein upon cell contact relieves riboregulator-mediated repression. This leads to a strong induction of the master regulator of T3SS/effector gene expression promoting an increase of the virulence potential and provokes a fast adaptation of the pathogen's fitness, e.g. to compensate for the imposed energetic burden.

## Introduction

Protein secretion plays a pivotal role in the interaction between pathogenic bacteria and their hosts. Pathogenic bacteria utilize different highly sophisticated secretion systems (Type I-VII) to translocate proteins across membranes into eukaryotic target cells in order to manipulate host cell functions and disrupt host homeostasis and immune defenses [1–3]. Of these, the type III secretion system (T3SS) is a key virulence factor in many plant, animal and human pathogenic bacteria that contributes to bacterial survival and colonization [1, 4]. T3SSs are contact-dependent secretion systems. An intimate contact between the pathogen and the eukaryotic target cell is the signal for the bacterium to induce expression of the secretion machinery and its secreted substrates, the so-called effector proteins, as well as the secretion procedure itself. T3SS gene expression and secretion is activated by growth conditions, certain chemicals, and environmental signals (temperature, pH, oxygen availability, host-associated signals) that mimic host cell contact, and by complex feedback control mechanisms [1, 2, 4]. One of the best-studied systems is the *Yersinia* T3SS [5, 6].

All human pathogenic *Yersinia* species, *Y*. *pestis*, *Y*. *pseudotuberculosis* and *Y*. *enterocolitica*, share the ability to survive and proliferate under adverse conditions in lymphatic tissues of their mammalian hosts. The Ysc T3SS and the secreted and translocated effector proteins, the Yops, protect *yersiniae* from phagocytosis by professional phagocytes. All T3SS-associated genes are encoded on the common 70 kb virulence plasmid (pYV/pCD1) [7, 8]. The T3SS forms a complex apparatus of about 20–25 proteins—the injectisome—that functions as a molecular syringe to inject bacterial effector proteins into targeted host cells [6, 8, 9]. Pathogenic *yersiniae* use the T3SS to secrete and translocate seven Yop effector proteins mainly into neutrophils and macrophages during infection to facilitate phagocytic avoidance, promote systemic spread, manipulate the inflammatory responses and control cell death programs [5, 10–12]. Translocation of Yops and expression of the T3SS/*yop* genes is strictly regulated and substantially increased after the bacteria established contact with their target cells [13, 14]. Transcription of the *Yersinia* T3SS/*yop* genes is intimately coupled to the activity of the T3 secretory machinery, i.e. transcription is strongly activated when the machinery is active. Growth under Ca^2+^-limiting conditions at 37°C mimics the host cell contact, a phenomenon known as the low calcium response (LCR), and is commonly used as a surrogate for the contact signal to convert the injectisome to a secretion competent state [15]. However, the underlying mechanisms of host cell contact-induced signal transduction and T3SS/*yop* induction remains enigmatic.

Some studies reported that YopD, a pivotal structural component of the translocation pore for direct delivery of Yop effectors into the target cells [16–19], acts as a sensing device for cell contact and is a negative regulator of T3SS/Yop in the absence of an inductive signal [17, 20–24]. It is further known that (i) loss of YopD causes an increase of the virulence plasmid copy number of pYV [25], and/or (ii) that YopD interacts with untranslated leader segments of T3SS/*yop* transcripts and blocks T3SS/Yop synthesis [22, 23]. These data suggested to us that YopD might be crucial for host cell contact-dependent T3SS/*yop* induction. To gain a better understanding of the underlying molecular mechanism, we first identified regulatory components implicated in triggering effector secretion upon cell contact and characterized how YopD interferes with this process. This revealed a novel virulence strategy in which a pathogen uses a translocator protein (YopD) to coopt important global riboregulators to control the master regulator (LcrF) of T3SS/effector genes (*ysc/yops*) in response to host cell contact.

## Results

### Host cell contact-mediated induction of the Ysc/Yop T3SS occurs through LcrF

**Fig 1:**
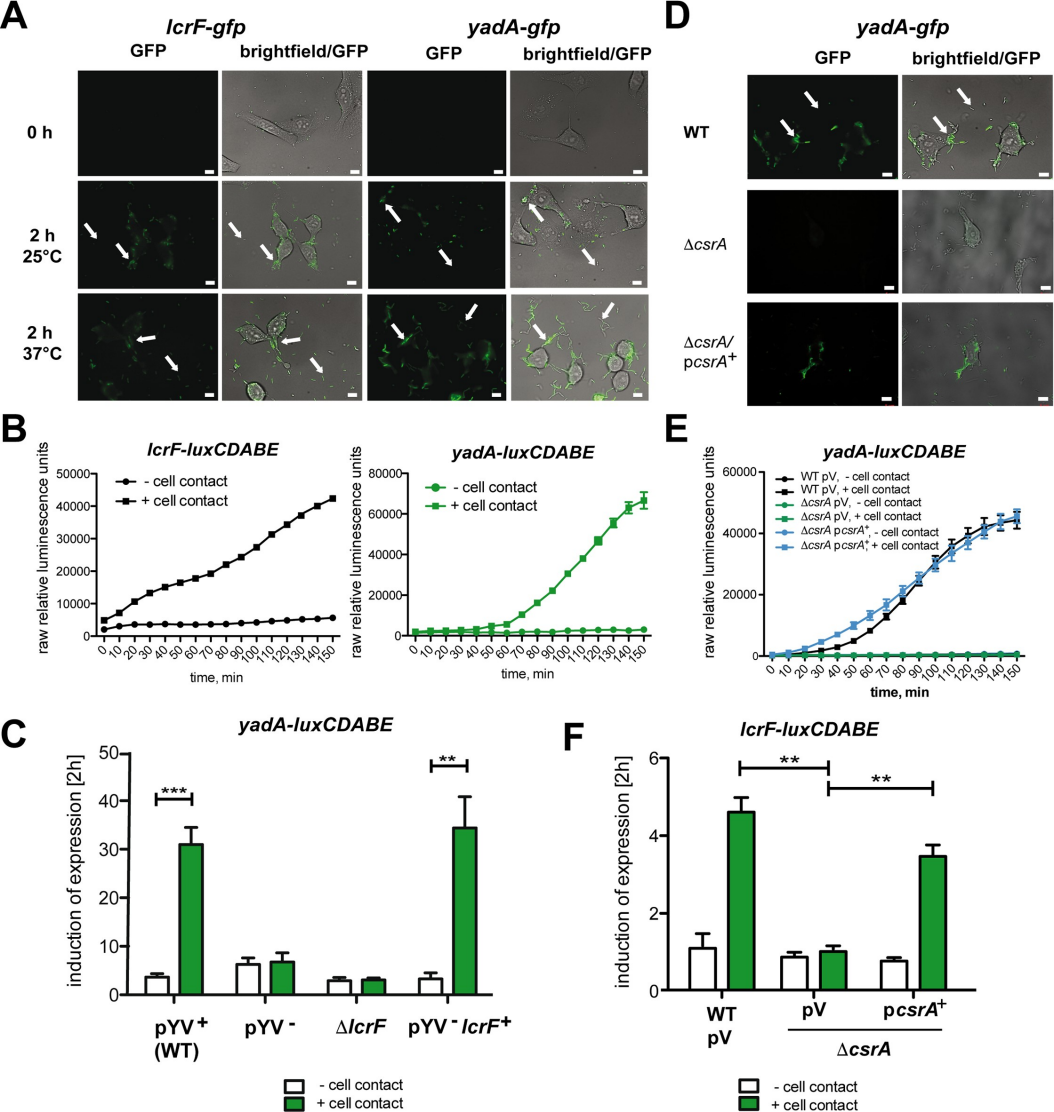
CsrA-dependent, host cell contact-mediated induction of *yadA* and *lcrF* expression. (A) HEp-2 cells infected with *Y*. *pseudotuberculosis* wildtype strain YPIII harboring a plasmid-encoded *yadA-gfp* (pJE9) or a *lcrF-gfp* fusion (pWO3) were incubated for at least 2 h at 25°C or 37°C. Arrows indicate bacteria in contact and without contact to host cells. White bars indicate 5 μm. (B) Strain YPIII harboring a plasmid-encoded *yadA-luxCDABE* (pWO41) or a *lcrF-luxCDABE* fusion (pWO42) were incubated with or without HEp-2 cells for 2.5 h at 25°C. Bioluminescence of the samples was monitored every 10 min to determine the kinetics of host cell contact-dependent expression of the reporter fusions. (C) Strains YPIII (WT), YP12 (pYV^-^), YP66 (Δ*lcrF*) and YP155 (pYV^-^, *lcrF*^+^) harboring the *yadA-luxCDABE* fusion plasmid pTS31 were used to infect HEp-2 cells or were incubated in PBS incubated without cells at 25°C. Bioluminescence of the samples was monitored after 2 h. The data represent the mean ± SEM of the fold change (end/start) from three independent biological replicates and were analyzed with Student’s t-test. **: P<0,01, ***: P<0,001. (D) HEp-2 cells infected with YPIII (WT), YP53 (Δ*csrA*) and YP53 pAKH56 (*csrA*^+^) harboring a *yadA-gfp* fusion (pJE9) were incubated for 4 h at 25°C. Arrows indicate bacteria with and without contact to host cells. White bars indicate 5 μm. (E) Strains YPIII pV (empty cloning vector pHSG576), YP53 (Δ*csrA*) pV and YP53 pKB60 (*csrA*^+^) harboring a plasmid-encoded *yadA-luxCDABE* (pWO41) were incubated with or without HEp-2 cells at 25°C. Bioluminescence of the samples was monitored every 10 min to determine the kinetics of host cell contact-dependent expression of the reporter fusions. (F) Strains YPIII, YP53 (Δ*csrA*) pV (empty cloning vector pHSG576) and YP53 pKB60 (*csrA*^+^) harboring a plasmid-encoded *lcrF-luxCDABE* (pWO42) were applied to infected HEp-2 cells or incubated in PBS without cells at 25°C. Bioluminescence of the samples was monitored after 2 h. The data represent the mean ± SEM of the fold change (end/start) from three independent biological replicates and were analyzed with Student’s t-test. **: P<0,01.

Host cell contact has previously been reported to be a potent signal that triggers synthesis of Yop effector proteins of *Y*. *pseudotuberculosis* [14, 26, 27]. To address whether host cell induction is promoted through upregulation of the main T3SS/Yop regulator LcrF, we monitored expression of the *lcrF* and the LcrF-dependent adhesin *yadA* fused to *gfp* or *luxCDABE* in bacteria infecting human epithelial cells. Cell-associated bacteria were characterized by a strong induction of the reporter fusions upon cell contact at 37°C, but also at 25°C when *ysc/yop* and *yadA* are usually fully repressed (Fig 1A and 1B). In agreement, a strong increase of LcrF and YadA levels were detectable in bacteria bound to host cells (S1A Fig). In contrast, no or only a very weak signal was detectable from bacteria attached to the wells (Fig 1A and 1B), or when other control fusions were used (S1B Fig).

Immediate contact-triggered induction of *lcrF* expression and delayed upregulation of *yadA* (Fig 1B) suggested that cell contact induction occurs through induction of *lcrF*. In fact, induction of *yadA* upon host cell contact failed in a Δ*lcrF* mutant and in a virulence plasmid-cured strain (pYV^-^) (Figs 1C and S1C). Host cell-contact induction could be reconstituted by integration of the *yscW*-*lcrF* locus into the *Y*. *pseudotuberculosis* chromosome in the absence of pYV. This indicated that one or more chromosomally-encoded regulator(s) are implicated in cell contact-triggered *lcrF* induction.

### The carbon storage regulator CsrA is required for cell contact-mediated *lcrF* expression

Our previous studies revealed that the pYV-encoded *yadA* and T3SS/*yop* genes are differentially regulated in the absence of the carbon storage regulator CsrA [28, 29]. This implied that their common regulator LcrF is likely the target for CsrA regulation. CsrA is a regulatory RNA-binding protein, which together with one or more non-coding RNAs (in *Yersinia* named CsrB and CsrC) constitute the Csr/Rsm system. This post-transcriptional control system regulates a large set of target transcripts involved in diverse physiological functions, ranging from virulence, metabolism and growth to motility, chemotaxis and stress resistance [30–33].

To address whether CsrA is implicated in host cell contact-mediated induction of pYV-encoded virulence factors, we monitored expression of *lcrF* and the LcrF-dependent *yadA* gene in a *csrA* mutant in response to host cell contact. As seen in Figs 1D, 1E and S2A host cell contact induction of *yadA* was abolished in the absence of the *csrA* gene but could be complemented by introduction of a *csrA*^+^ plasmid. A similar expression pattern was found for the *lcrF* gene (Fig 1F), indicating that CsrA is essential for cell contact-mediated LcrF induction.

### CsrA activates translation and increases stability of the *lcrF* transcript under secretion condition

**Fig 2:**
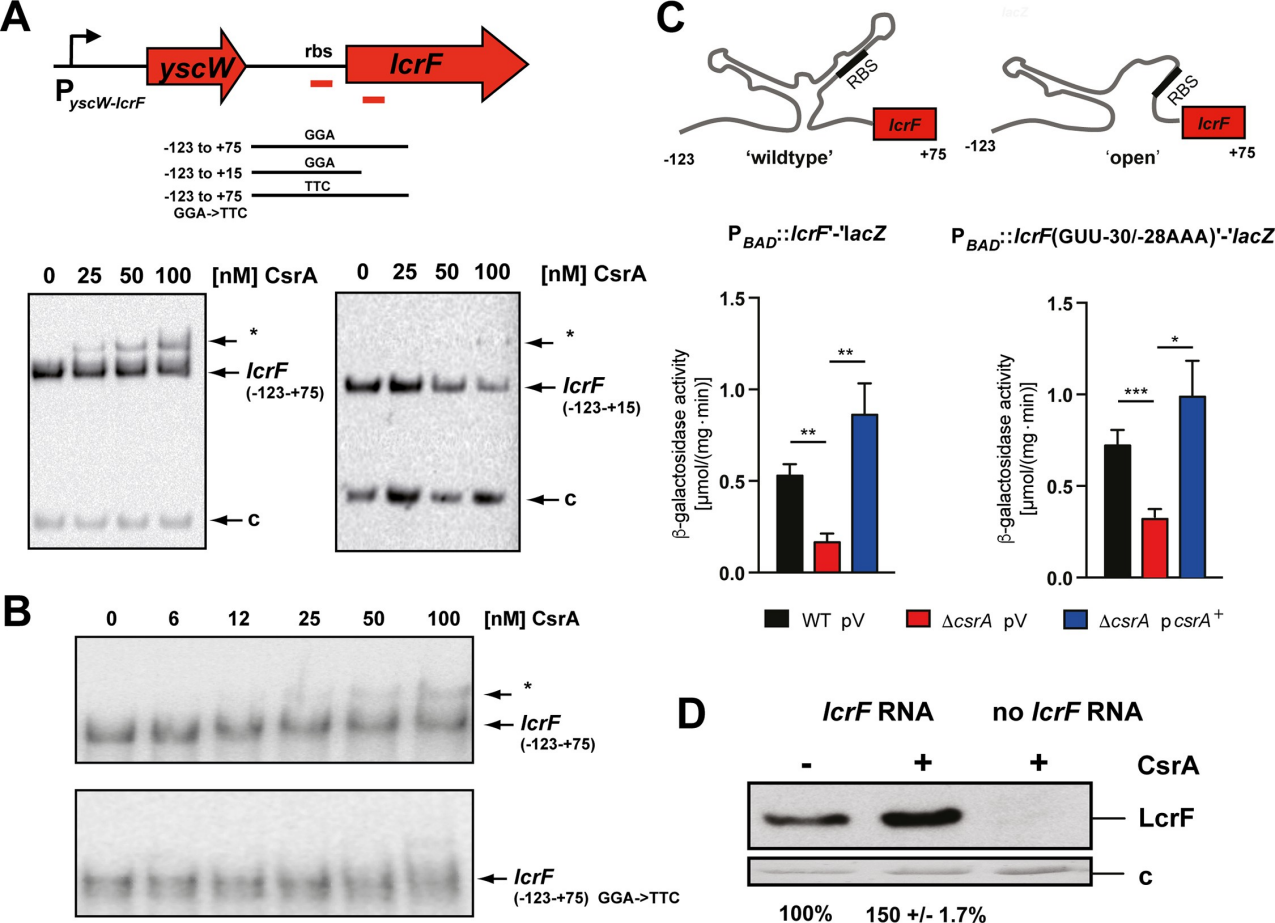
Influence of CsrA on *lcrF* transcript translation. (A) Upper panel: Overview of the *yscW-lcrF* locus and the generated *lcrF* transcripts used for the RNA binding assay below and in (B); red bars: CsrA binding sites. The *yscW-lcrF* promoter is indicated by a broken arrow; the identified CsrA binding sites are indicated with red bars. The numbers give the nucleotide positions with respect to the translation initiation site of *lcrF*. Lower panel: RNA binding assays with increasing concentration of purified CsrA-His_6_ to *lcrF* transcripts harboring the region of position -123 to +75 (left panel) or -123 to +15 (right panel) with respect to the translational start site. The CsrA-*lcrF* mRNA complex is indicated by a star. c: *hns* control transcript. (B) The *lcrF* transcript (-123 to +75) and a mutant transcript in which the GGA motif is exchanged against TTC were incubated with increasing concentration of purified CsrA-His_6_. The CsrA-*lcrF* mRNA complex is indicated by a star. (C) To study CsrA influence on translation initiation of *lcrF*, expression of an arabinose inducible translational *lcrF-lacZ* fusion encoded on pKB14 and a mutant variant with an open *lcrF* RNA thermometer element encoded on pKB99 was analyzed in YPIII (WT) pV (empty cloning vector pHSG576) and YP53 (Δ*csrA*) pV and YP53 pKB60 (*csrA*^+^). The bacteria harboring the different *lcrF-lacZ* fusions were grown at 25°C to exponential phase and were then shifted for 4 h to 37°C in the presence of 0.1% arabinose and absence of Ca^2+^. β-galactosidase activities represent the mean ± SD from three independent biological replicates which were analyzed with Student’s t-test. *: P<0,05: **: P<0,01, ***: P<0,001. (D) An amplified *lcrF* DNA fragment of pMP1 was used for *in vitro* transcription. The resulting *lcrF* mRNA fragment was used for *in vitro* translation in the absence or presence of CsrA. Samples were analyzed by western blotting with a polyclonal antibody directed against LcrF. An assay without the *lcrF* template RNA was used as negative control. c: loading control (lower panel).

CsrA is able to post-transcriptionally activate expression of its target genes by binding to the leader transcript to (i) disrupt an RNA structure that would otherwise block the RBS or (ii) prevent transcript degradation by RNases [31, 32]. To further investigate the influence of CsrA on LcrF synthesis, we first tested whether CsrA binds directly and specifically to the 5’- UTR of the *lcrF* transcript. This region forms a thermo-sensitive secondary structure of two stem-loops, which sequesters the *lcrF* ribosomal binding site by a stretch of four uracils (fourU RNA thermometer). Opening of this structure is favored at 37°C and permits ribosome binding at host body temperature [34]. As shown in Fig 2A (left panel), incubation of a *lcrF* transcript (extending from -123 to +75 with respect to the *lcrF* start codon) and an *hns* control transcript with increasing concentrations of purified CsrA-His_6_ resulted in the formation of a higher molecular weight complex of the *lcrF* transcript, but not of the *hns* transcript. The shifted *lcrF* leader transcript included one primary CsrA binding site within the RBS of the *lcrF* gene (AGGA) and a secondary binding site (AGAGA) shortly downstream of the start codon. The secondary binding site is required for CsrA interaction, as a fragment exhibiting a 60 nt deletion, eliminating the potential secondary binding site caused a drastic reduction of CsrA-*lcrF* complex formation (Fig 2A, right panel). A very small amount of CsrA-*lcrF* complexes was only observed at high CsrA concentrations (≥100 nM), consistent with a much lower binding affinity to the incomplete binding site. To confirm the postulated primary CsrA binding site, we also exchanged the GGA motif within the RBS to TTC. We found that introduced mutations abolished or strongly reduced CsrA interaction with the *lcrF* transcript (Fig 2B). Only a very weak band representing the CsrA-*lcrF* complex was found at high CsrA concentrations (≥100 nM) most likely due to CsrA binding to the secondary site.

To further prove whether CsrA interaction with the *lcrF* leader improves translation initiation, we first tested the expression of a translational P_*BAD*_::*lcrF’-‘lacZ* fusion harboring only the *lcrF* 5’-UTR to exclude CsrA influence on *lcrF* transcription. As shown in Fig 2C (left panel), loss of CsrA reduced expression of the P_*BAD*_::*lcrF’-‘lacZ* fusion, and ectopic expression of the *csrA* gene on a plasmid restored expression to wildtype levels. As the RBS of the *lcrF* gene is part of an RNA thermometer [34], it is possible that CsrA is required to promote and/or support thermo-induced opening of the stem-loop structure at moderate temperature to allow ribosome access. To investigate the influence of CsrA on the accessibility of the RBS, we also tested the effect of CsrA on a derivative of the P_*BAD*_::*lcrF’-‘lacZ* fusion harboring nucleotide exchanges (GUU-30/-28AAA) that lead to a more 'open' conformation of the thermosensing RNA element (mimicking host temperature) in which the RBS is more accessible [34]. As shown in Fig 2C (right panel), the overall synthesis of the LcrF-LacZ fusion protein was increased, but CsrA was still required for maximal induction of LcrF-LacZ synthesis. This indicated that CsrA binding in the vicinity of the start codon enhances initiation of LcrF synthesis. In fact, *in vitro* translation assays using a *lcrF* RNA template showed that addition of CsrA enhanced LcrF synthesis (Fig 2D).

**Fig 3:**
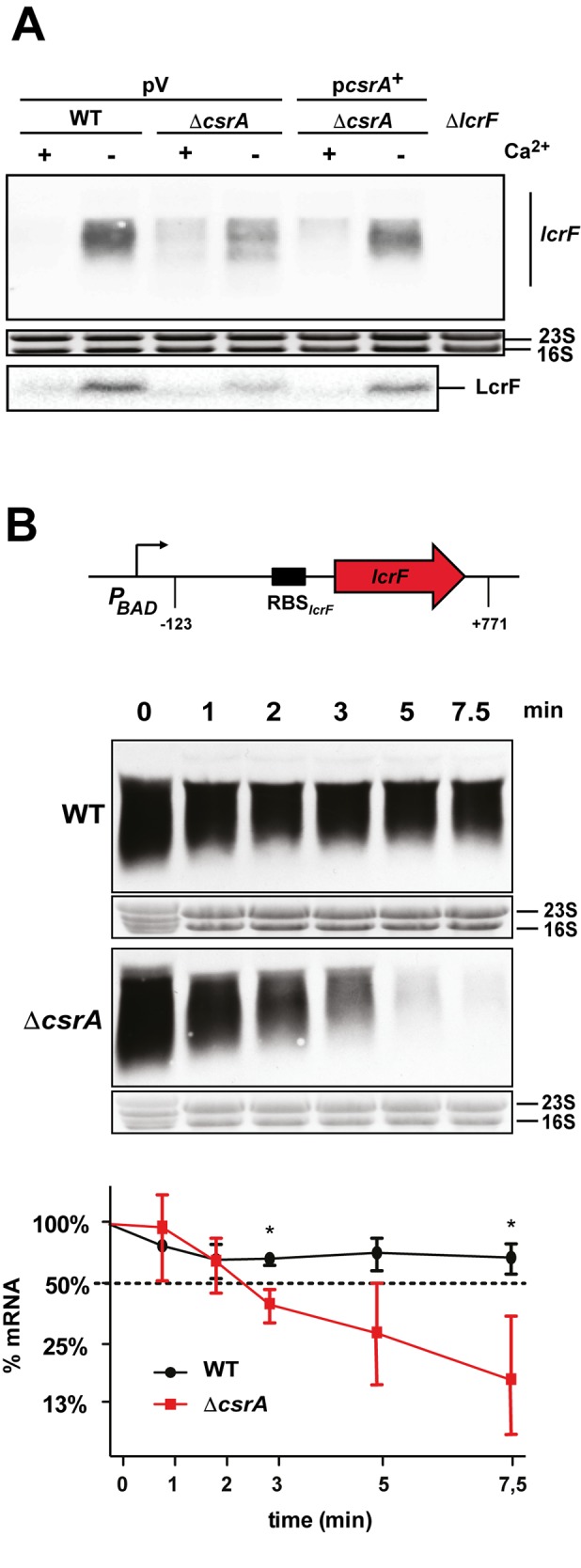
Influence of CsrA on *lcrF* transcript stability. (A) *Y*. *pseudotuberculosis* strains YPIII (WT), and YP53 (Δ*csrA*) harboring the empty vector pV (pHSG576) or p*csrA*^+^ (pKB60) were grown to exponential phase (OD_600_ of 0.5) at 25°C and then shifted to 37°C in the presence (+) or absence (-) of Ca^2+^ by addition of 20 mM sodium oxalate and 20 mM MgCl_2_. After growth for additional 4 h total RNA of the non-induced and induced cultures was prepared and subjected to northern blotting using a *lcrF* specific probe (upper panel). In parallel, LcrF protein levels were assessed by western blotting. Whole cell extracts were prepared and separated by 15% Tricine-SDS polyacrylamide electrophoresis. The LcrF and the H-NS protein were detected by immunoblotting with polyclonal anti-LcrF or anti-H-NS antibodies. (B) YPIII and YP53 (Δ*csrA*) harboring a P_*BAD*_::*lcrF* (pMP1) expression plasmid were grown to exponential phase (OD_600_ of 0.5) at 25°C. The cultures were shifted to 37°C, depleted of Ca^2+^ and grown for additional 4 h before rifampicin was added to the cultures to stop transcription. Samples of the cultures were taken directly (0 min) and 1, 2, 3, 5 and 7.5 min after rifampicin treatment. Total RNA of the samples was prepared and subjected to northern blotting using a *lcrF* specific probe. The 16S and 23S rRNAs served as loading controls (upper panel). The *lcrF* transcript levels were quantified and plotted to determine the mRNA degradation rate (lower panel). The intensity for the 0 min sample was set to 100%, and other samples were normalized to time point 0 sample. The data represent the mean ± SD from three independent biological replicates analyzed with Student’s t-test. *: P<0,05.

In parallel, we tested whether CsrA-mediated activation of *lcrF* mRNA translation has an influence on *lcrF* transcript and protein levels under T3SS/Yop-inducing and non-inducing conditions (+/-Ca^2+^). As shown in Fig 3A, we found that the amount of the *lcrF* mRNA, as well as the LcrF protein, is significantly reduced in the *csrA* mutant under secretion conditions. To test whether the *lcrF* transcript is less stable in the absence of the riboregulator, we determined the stability of the *lcrF* mRNA. To exclude transcriptional control and changes of the virulence plasmid copy number [25], we expressed the *lcrF* gene under control of the P_*BAD*_ promoter. Absence of CsrA resulted in an approximately two-fold reduction of the full-length *lcrF* mRNA within 2.5 min after inhibition of transcription by rifampicin, whereas the *lcrF* transcript was stable in the presence of CsrA (Fig 3B). In summary, our analysis revealed that CsrA interacts with the 5’-UTR of the *lcrF* mRNA to promote translation initiation and that CsrA is required to stabilize the *lcrF* transcript.

### T3SS/Yop-inducing conditions manipulate expression of the Csr system

**Fig 4:**
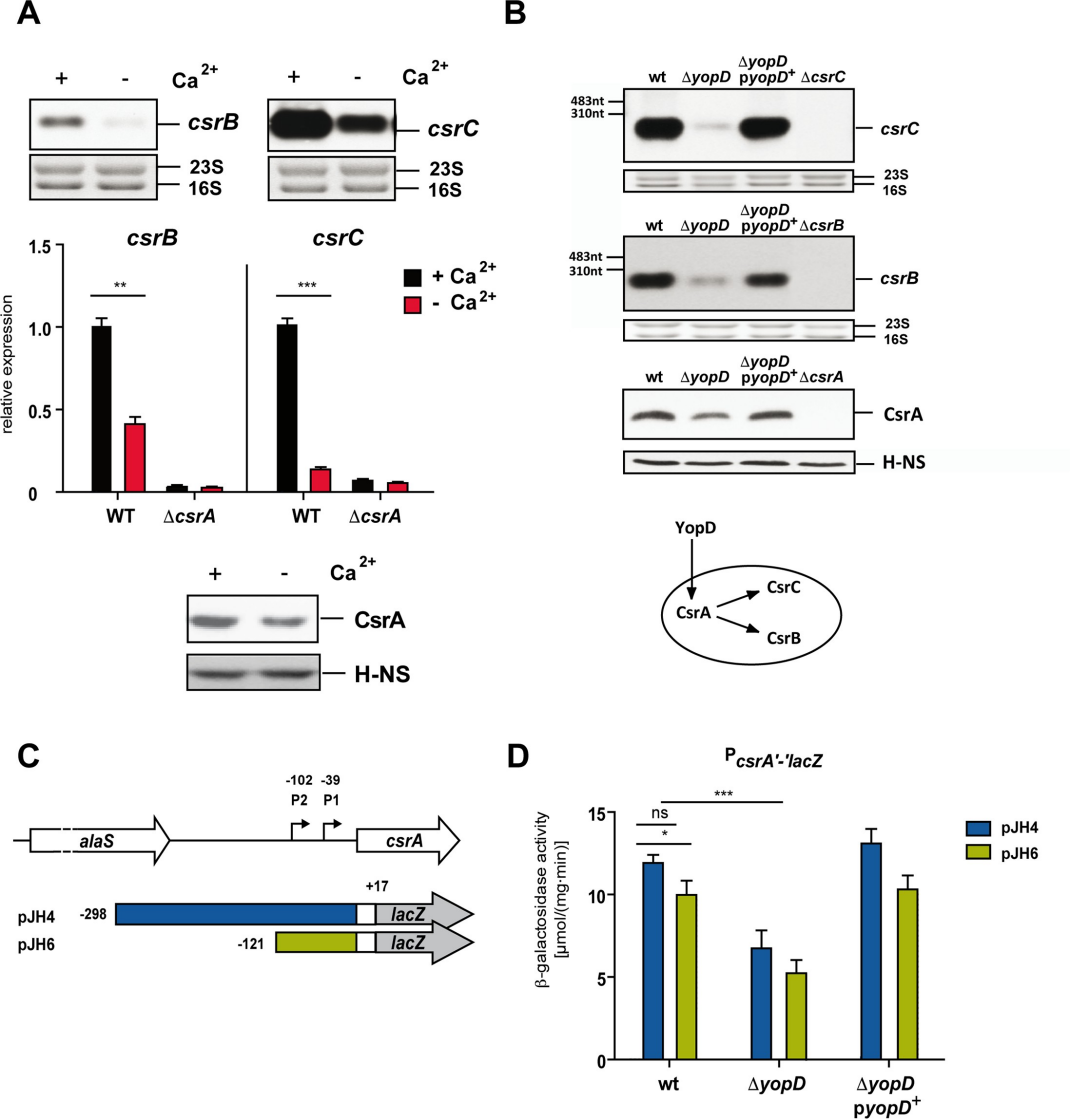
T3SS/Yop-inducing conditions and YopD manipulate expression of the Csr system. (A) *Y*. *pseudotuberculosis* strains YPIII (WT) and YP53 (Δ*csrA*) were grown to exponential phase (OD_600_ of 0.5) at 25°C and then shifted to 37°C in the presence (-) or absence of Ca^2+^ (-) by addition of 20 mM sodium oxalate and 20 mM MgCl_2_. After growth for additional 4 h total RNA of the non-induced and induced cultures of WT and the Δ*csrA* strain was prepared. Upper panel: Total RNA of the WT strain was subjected to northern blotting using *csrB-* and *csrC-*specific probes. Middle panel: A real-time qRT-PCR analysis was performed with total RNA of YPIII (WT) and YP53 (Δ*csrA*) with specific primers for the *csrB* and *csrC* transcripts. Transcript levels were normalized to the *sopB* reference transcript and are shown as relative expression to the wildtype (YPIII) under non-secretion (+Ca^2+^) and secretion (-Ca^2+^) conditions. Data represent the mean ± SD of three biological and two technical replicates analyzed with a one sample t-test with **, P < 0.01, and ***, P < 0.001. Lower panel: Whole cell extracts of the cultures were separated by SDS-polyacrylamide electrophoresis and CsrA and H-NS proteins were detected by western blot analysis with polyclonal anti-CsrA or anti-H-NS (control) sera. (B) *Y*. *pseudotuberculosis* strains YPIII (WT) pRS1 (empty vector), YP91 (Δ*yopD*) pRS1, YP91 (Δ*yopD*) pRS2 (*yopD*^+^) were grown under non-secretion conditions (+Ca^2+^). Total RNA of the cultures was prepared and subjected to northern blotting using *csrB-* and *csrC-*specific probes. The 16S and 23S rRNAs served as loading controls. Whole cell extracts of the identical non-induced and induced cultures were separated by SDS polyacrylamide electrophoresis and subjected to western blotting using a polyclonal anti-CsrA antibody. H-NS detected by a polyclonal antibody was used as loading control. Lower panel: scheme of the regulatory influence of YopD on the Csr systems components. (C) Schematic overview of the transcriptional *csrA-lacZ* reporter fusions encoded by pJH4 and pJH6 harboring different fragments of the *csrA* promoter region including the two identified promoters P1 and P2. The numbers indicate the 5’-end of the *csrA* upstream region fused to *lacZ* with respect to the translational start site of the *csrA* gene. (D) Strains YPIII (WT), YP91 (Δ*yopD*) pRS15 (empty vector), and YP91 (Δ*yopD*) pRS16 (*yopD*^+^) harboring the different indicated *csrA-lacZ* reporter plasmids were grown to late exponential phase at 37°C. β-galactosidase activity of the different cultures was monitored. The data represent the mean ± SD from at least three independent biological replicates performed in triplicates and were analyzed with Student’s t-test; *: P<0,05, ***: P<0,001, n.s.: P>0.05.

Since CsrA induces LcrF upon host cell contact in a post-transcriptional manner, we investigated whether Yop secretion-inducing conditions influence the amount and/or ratio of the Csr system components, i.e. the RNA-binding regulator CsrA and the counteracting regulatory RNAs CsrB and CsrC. To test this, we used Ca^2+^ limiting conditions as a substitute for host cell contact to induce Yop secretion. As shown in Fig 4A, significantly lower transcript levels of the CsrB and CsrC RNAs components were detectable in the bacterial cell upon Ca^2+^-depletion, whereas the overall amount of CsrA was only slightly reduced, indicating that more non CsrB/C-bound and hence more active CsrA is available under secretion conditions. These data suggest that a regulatory circuit is operative in which the release of the Yops influences Csr function to control LcrF and thus T3SS/Yop synthesis.

### The translocon protein YopD controls the Csr system

Several studies demonstrated that expression of the T3SS/*yop* genes is under negative feedback control implicating the translocator protein YopD [17, 20, 22, 35]. Therefore, we investigated whether export and removal of YopD from the bacterial cytoplasm is responsible for downregulation of the Csr components under Yop secretion-inducing conditions (Figs 4B and S3). We found that a *yopD* deficient strain is characterized by a strong reduction of the CsrB and CsrC sRNAs levels and caused a small decrease of the intracellular CsrA protein levels (Fig 4B), similar to what has been observed under Ca^2+^ depletion (Fig 4A, lower panel).

CsrA was shown to indirectly inhibit the degradation of the CsrB and CsrC sRNAs in *E*. *coli*, [36, 37], and also in *Yersinia* CsrA is required to maintain CsrB and CsrC (Figs 4A, middle panels, S3). This suggested that YopD influence on all the Csr components occurs through regulation of CsrA (Fig 4B, lower panel).

**Fig 5:**
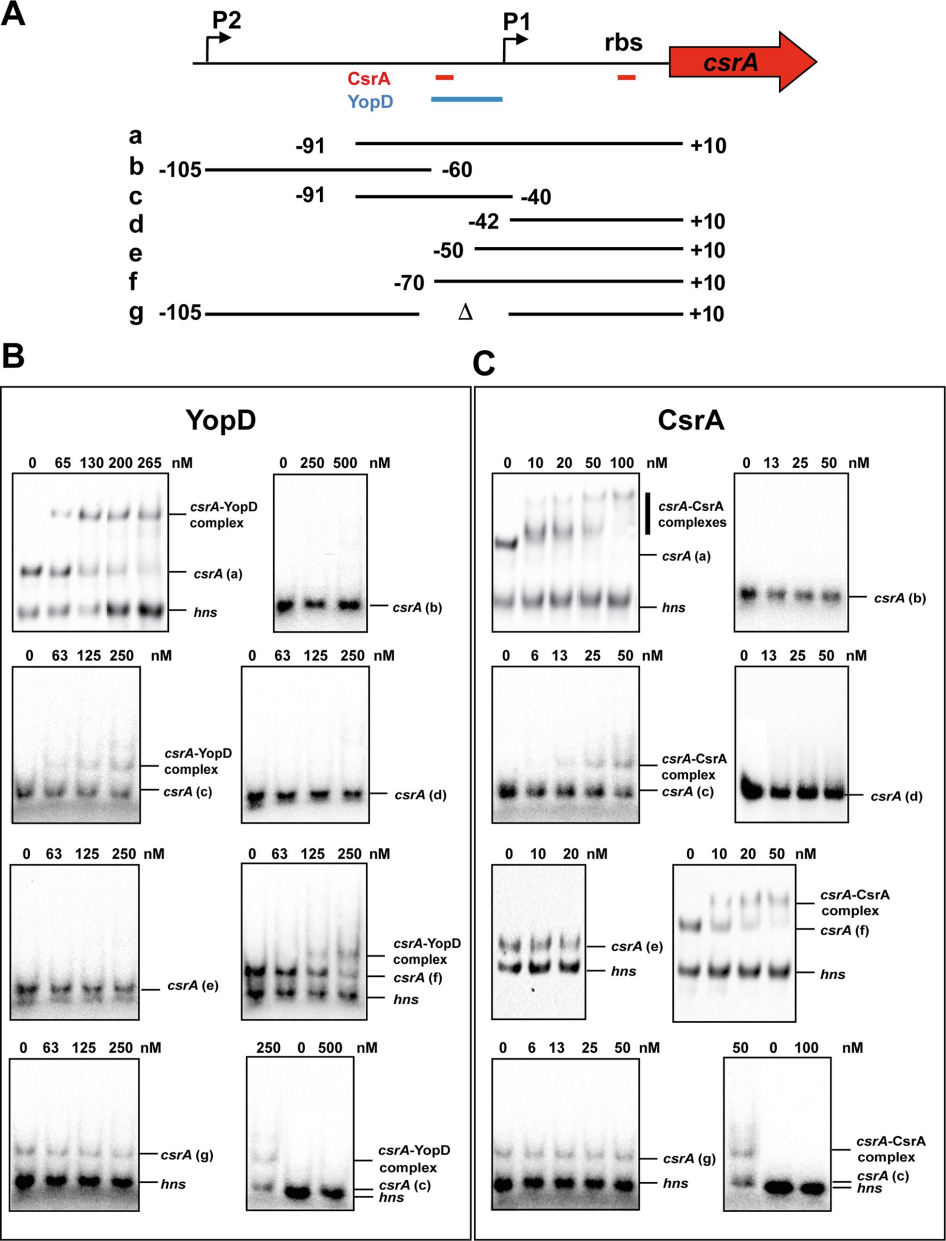
Interaction of CsrA and YopD with the *csrA* transcript. (A) Schematic overview of the *csrA* locus and the generated *csrA* transcript fragments used for the RNA binding assay in B-C. The two promoters identified by a previous RNA-Seq analysis [38] are indicated by broken arrows. The identified CsrA and YopD binding sites are indicated with red and blue bars. The numbers indicate the nucleotide positions with respect to the translation initiation site of *csrA*. The alphabetic characters indicate the transcripts used in B-C. (B) The *in vitro* generated transcript (a) was incubated with increasing amounts (left panel) and transcripts (b)-(d) were incubated with a defined (650 nmol, right panel) amount of purified YopD. The *csrA*-YopD complex is indicated. c: The *hns* transcript was used as negative control. (C) The *in vitro* generated transcripts a (left panel), f and e (right panel) were incubated with increasing amounts of purified CsrA. The *csrA*-CsrA complex is indicated. c: The *hns* transcript was used as negative control.

To further test this hypothesis, we analyzed expression of two translational *csrA'-'lacZ* fusions of which one harbored a deletion of the *csrA* upstream region which eliminated one of the two active promoters determined in previous studies [28, 38] (Fig 4C). The deletion of upstream promoter P2 led only to a slight reduction of *csrA'-'lacZ* expression, demonstrating that P1 is the most active promoter. Absence of YopD reduced the overall activity of both reporters to about 50%, indicating that YopD-mediated activation involves sequences located downstream of position -121 (Fig 4C and 4D). Next, we tested whether purified YopD is able to recognize and bind to the 5’-UTR of *csrA* using transcripts covering different portions of the 5’-UTR downstream of promoter P2 (Fig 5A). We detected specific binding of YopD only to *csrA* RNA fragments harboring sequences from position -60 to -40 with respect to the start codon of *csrA* (Fig 5B).

The inspection of the *csrA* 5’-UTR further revealed two potential primary CsrA binding motifs (i.e. ANGGA sequences), one of which overlaps the RBS of the *csrA* transcript (Fig 5A). This suggested that *csrA* translation is negatively autoregulated, by directly competing with 30S ribosomal binding, similar to what has been shown for *E*. *coli csrA* [39]. RNA-binding shift assay with an *in vitro csrA* transcript harboring the 5’-UTR sequences from position -70 to +10 revealed the formation of *csrA*-CsrA complexes, but a 5’-deletion to position -50, which eliminates one primary CsrA binding site, abolished CsrA-*csrA* complex formation (Fig 5C). This strongly suggests that CsrA binding to ANGGA motifs in the vicinity of promoter P1 and the RBS builds a translational repressing complex. As one CsrA binding site overlaps with the identified YopD binding region, we postulate that YopD-dependent regulation of the Csr system occurs through interference with *csrA* autoregulation. YopD binding to the *csrA* transcript might prevent formation of the higher-order CsrA-RNA complex and allows free access of the 30S ribosomal subunit, which stimulates CsrA synthesis from the P2 initiated transcript.

### Presence of YopD enhances *lcrF* transcript degradation under non-secretion conditions

**Fig 6:**
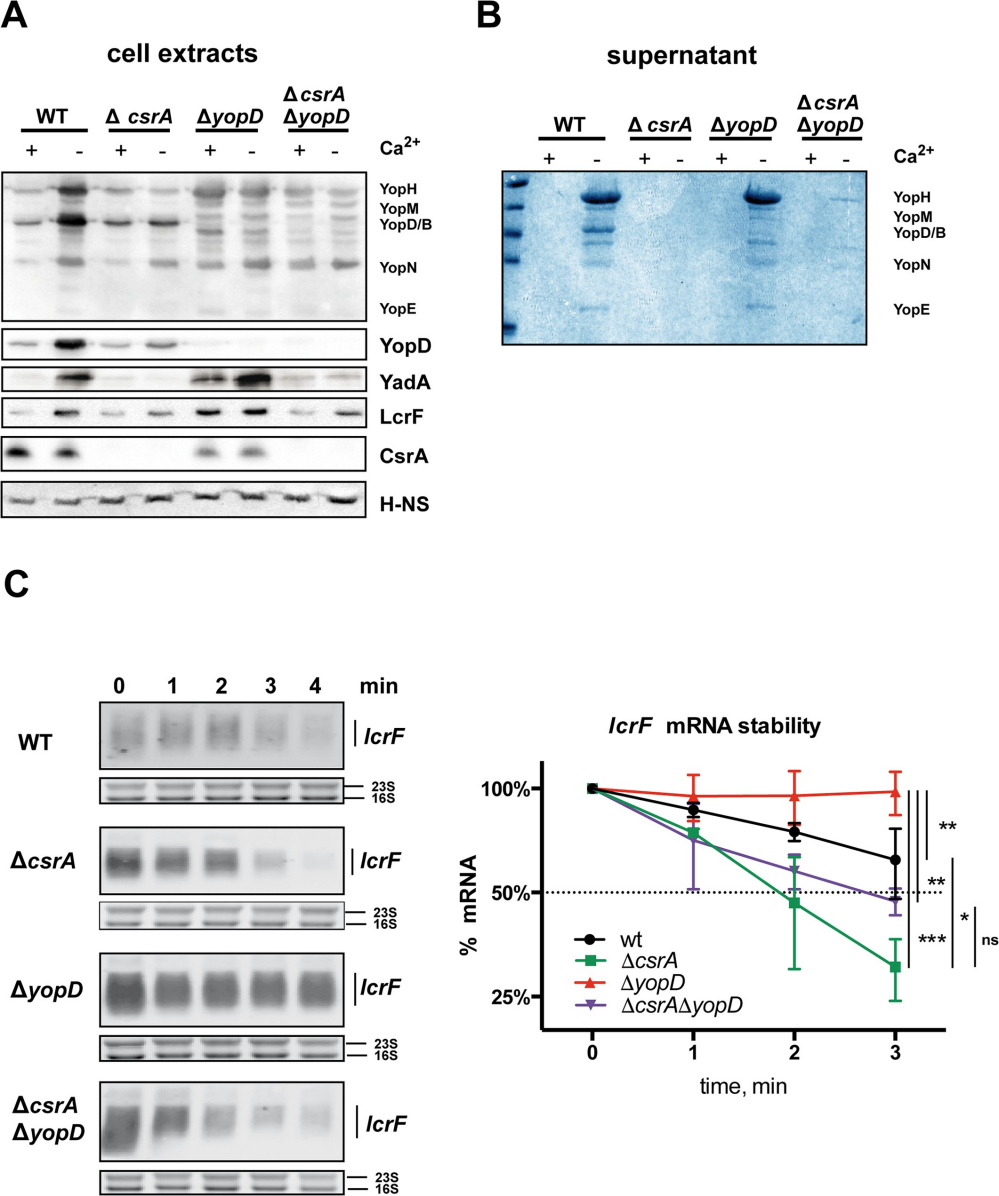
Influence of CsrA and YopD on the expression of Yops, YadA and LcrF. Strains YPIII, YP53 (Δ*csrA*), YP91 (Δ*yopD*), and YP145 (Δ*csrA*, Δ*yopD*) were grown in LB at 37°C in the absence (non-secretion conditions) or presence of 20 mM MgCl_2_ and 20 mM sodium oxalate for 4 h (secretion conditions). (A) Whole cell extract of the bacterial cultures were prepared and analyzed by western blotting with polyclonal anti-Yop, anti-YadA, anti-LcrF, anti-YopD or anti-CsrA antibodies. H-NS detected with polyclonal anti-H-NS served as loading control. (B) In parallel, proteins in the supernatant were precipitated with TCA, separated by SDS polyacrylamide electrophoresis and stained with Coomassie blue. A *lcrF* mutant was used as control. (C) To determine the stability of the *lcrF* mRNA rifampicin was added to cultures of YPIII, YP53 (Δ*csrA*), YP91 (Δ*yopD*), and YP145 (Δ*csrA*, Δ*yopD*) grown at 37°C to stop transcription. Samples of the cultures were taken directly (0 min) and 1, 2, 3, and 5 min after rifampicin treatment. Total RNA of the samples was prepared and subjected to northern blotting using an *lcrF* specific probe. The 16S and 23S rRNAs served as loading controls. A representative of at least three independent experiments is presented (left panel). The *lcrF* transcript levels were quantified using the ImageJ software and plotted to determine the mRNA degradation rate (right panel). The band intensity for the 0-min sample was set to 100%, and other samples were normalized to the 0-min sample. The data represent the mean ± SD from three independent biological replicates. The end point was analyzed using ANOVA with Dunnetts post-test for multiple comparisons. *: P<0,05, **: P<0,01; ***: P<0.001; ns: not statistically significant.

To further characterize the YopD-CsrA interplay, we compared the synthesis of LcrF and the LcrF-dependent Yops and YadA in the absence of CsrA and/or YopD. LcrF, YadA and Yop synthesis, as well as Yop secretion, were abolished in the Δ*csrA* strain under Ca^2+^-limiting conditions and host cell contact, as expected from our previous results, demonstrating that CsrA activates LcrF synthesis (Figs 1D–1F and 6A and 6B,). In contrast, a strong induction of LcrF, YadA and Yop expression was detected in the absence of YopD under non-secretion conditions. This is consistent with former studies, demonstrating that YopD acts as a negative regulator of T3SS/*yop* gene expression in the absence of an inductive signal [17, 20]. However, although lower amounts of the CsrA are produced in the *yopD* mutant, LcrF, Yop and YadA synthesis is not reduced compared to wildtype but enhanced. This indicated that YopD has an additional influence on *lcrF* expression, which is independent of CsrA.

To gain information about the individual contribution, we also tested how YopD influences LcrF, YadA and Yop synthesis as well as Yop secretion in the absence of CsrA. A significant reduction in the production levels and a failure of Yop secretion was also observed with the Δ*csrA/*Δ*yopD* double mutant, but the overall influence is not as severe as in the Δ*csrA* strain (Fig 6A and 6B). This indicated that YopD acts upstream of CsrA, but additional control mechanisms appear to exist by which YopD impacts LcrF synthesis independently of CsrA. We next tested how YopD affects *lcrF* transcript levels in the presence and absence of CsrA. RNA degradation assays revealed that the half-life of the *lcrF* transcript is considerably enhanced in the absence of *yopD*, but this stabilization of the *lcrF* mRNA is not apparent in the Δ*csrA*/Δ*yopD* mutant (Fig 6C). Rapid degradation of the *lcrF* transcript is observed, demonstrating that stabilization of the *lcrF* transcript is largely dependent on CsrA. However, slightly higher amounts of YadA and the Yop proteins were detectable in the Δ*csrA*/Δ*yopD* mutant compared to the Δ*csrA* strain, indicating that YopD does not solely affect these LcrF-activated virulence factors through regulation of CsrA.

### YopD interferes with components of the degradosome

**Fig 7:**
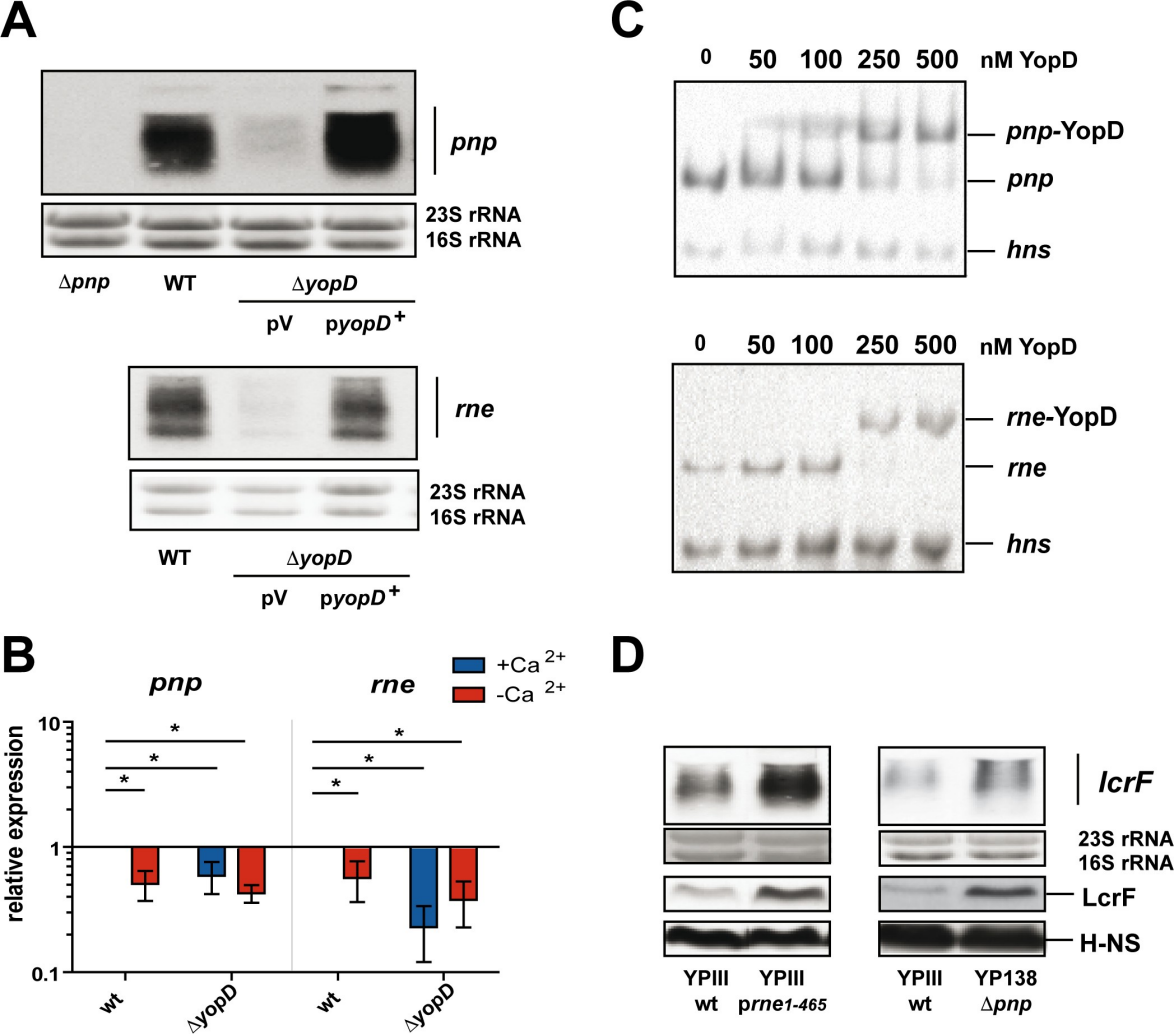
YopD influence on the degradosome RNases PNPase and RNase E. (A) *Y*. *pseudotuberculosis* strain YPIII (WT), YP91 (Δ*yopD*) pRS1 (empty vector), YP91 (Δ*yopD*) pRS2 (*yopD*^+^) and YP138 (Δ*pnp*) were grown at 37°C. Total RNA of the cultures was prepared and subjected to northern blotting using a *pnp* (upper panel) or *rne* (lower panel) specific probe. Total RNA of YP138 (Δ*pnp*) was used as negative control, and the 16S and 23S rRNAs served as loading controls. A representative of at least three independent experiments is presented. (B) *Y*. *pseudotuberculosis* strain YPIII (WT) and YP91 (Δ*yopD*) were grown at 37°C in the presence (+Ca^2+^, non-secretion conditions) or absence (-Ca^2+^, secretion conditions) of 20 mM MgCl_2_ and 20 mM sodium oxalate. Total RNA was prepared and a real-time qRT-PCR analysis was performed with specific primers for the *pnp* and *rne* transcripts. Transcript levels were normalized to the *sopB* reference transcript and are shown as relative expression to the wildtype (YPIII) under non-secretion (+Ca^2+^) conditions. Relative expression data of both mRNAs show the mean ± SD from at least three independent replicates and were analyzed with a one sample t-test with P-value: *: P<0,05. (C) The *in vitro* generated *pnp* (upper panel), *rne* (lower panel) and *hns* transcripts were incubated with increasing amounts of purified YopD. The *pnp- and rne*-YopD complexes are indicated. (D) *Y*. *pseudotuberculosis* strain YPIII (WT), YPIII p*rne*_1-465_ expressing a dominant negative *rne* variant (pRS40) and YP138 (Δ*pnp*) were grown at 37°C. Total RNA of the cultures was prepared and subjected to northern blotting using an *lcrF* specific probe. The 16S and 23S rRNAs served as loading controls. In parallel, whole cell extracts of the cultures were prepared and western blot analysis using a polyclonal antibody directed against LcrF and H-NS (control) was performed.

Since stabilization of the *lcrF* transcript in the absence of YopD was very prominent (Fig 6C), we hypothesized that this riboregulator could interfere with the RNA degradation machinery. To test this assumption, we first analyzed whether loss of YopD has an influence on the synthesis of the two RNases, PNPase and RNase E, which are components of the bacterial degradosome. As demonstrated by northern blotting and qRT-PCR analysis (Fig 7A and 7B), both *pnp* and *rne* transcript levels were significantly reduced in the *yopD* deletion strain under non-secretion conditions. Importantly, secretion alone (which eliminates YopD from the cytoplasm) is sufficient to reduce *pnp* and *rne* mRNA levels (Fig 7B). Moreover, we found that purified YopD is able to specifically interact with the 5'-UTR of the *pnp* and *rne* transcripts (Fig 7C). However, the binding affinity of YopD to the 5'-UTR regions, in particular to the *rne*, is rather low compared to the 5'-UTR of the *csrA* transcript, implying that the YopD-mediated influence occurs predominantly when the intracellular YopD concentration is increased. To further characterize the influence of YopD on *rne* and *pnp* expression we constructed *rne-lacZ* and *pnp-lacZ* transcriptional and translational fusion. As shown in S4 Fig, expression of both the *rne-lacZ* and *pnp-lacZ* translational fusion was significantly reduced in the absence of YopD. Expression of the *rne-lacZ* transcriptional fusion is also reduced to 69% in the absence of YopD. This confirmed that YopD activates the synthesis of RNase E, but it also indicated that this influence occurs mainly on the transcriptional level. In contrast, no influence of YopD is detectable with the *pnp-lacZ* transcriptional fusion. This would be expected when YopD predominantly influences PNPase synthesis on the post-transcriptional level. However, the overall expression of the *pnp-lacZ* fusion is very low, which makes the interpretation of the result difficult.

Based on the fact that YopD influenced *rne* and *pnp* transcript levels, we assumed that in the absence of host cell contact intracellular accumulating YopD could destabilize *lcrF* transcripts by upregulating PNPase and RNase E levels. To verify this assumption, we constructed a *pnp* mutant and a dominant-negative rne* (1–465) derivative (a *rne* mutant is lethal, [40]) and tested whether a decrease of functional degradosomes affects *lcrF* transcript and LcrF protein levels. As shown in Fig 7D, loss of PNPase or functional RNase E has a positive influence on LcrF synthesis as considerably higher amounts of the virulence regulator are detectable in the *pnp* mutant and the rne* expression strain. Taken together this demonstrated that YopD manipulates not only LcrF production by modulating CsrA levels, it also influences *lcrF* mRNA degradation by manipulating RNase E and PNPase synthesis.

## Discussion

Triggering of T3SS/effector gene expression upon host cell contact is a well-known hallmark of T3SS regulation in many important Gram-negative plant, animal and human pathogens; however, the molecular mechanism initiating this process is still unclear. Up to this point, a role of the translocon protein YopD in this process in *Yersinia* was assumed [17, 20], but how cell contact is transmitted and translated to trigger this response remained unclear. The translocon protein could have a passive role, e.g. by inducing conformational changes in components of the secretion needle upon translocon formation which then initiates a set of signaling pathways to modulate T3SS/Yop synthesis. Alternatively, YopD could actively control T3SS/*yop* gene expression. Our data demonstrate that YopD influences synthesis of the T3SS/Yop machinery and that this involves hijacking of multiple RNA regulators, the carbon storage regulator system and the degradosome components RNase E and PNPase.

**Fig 8:**
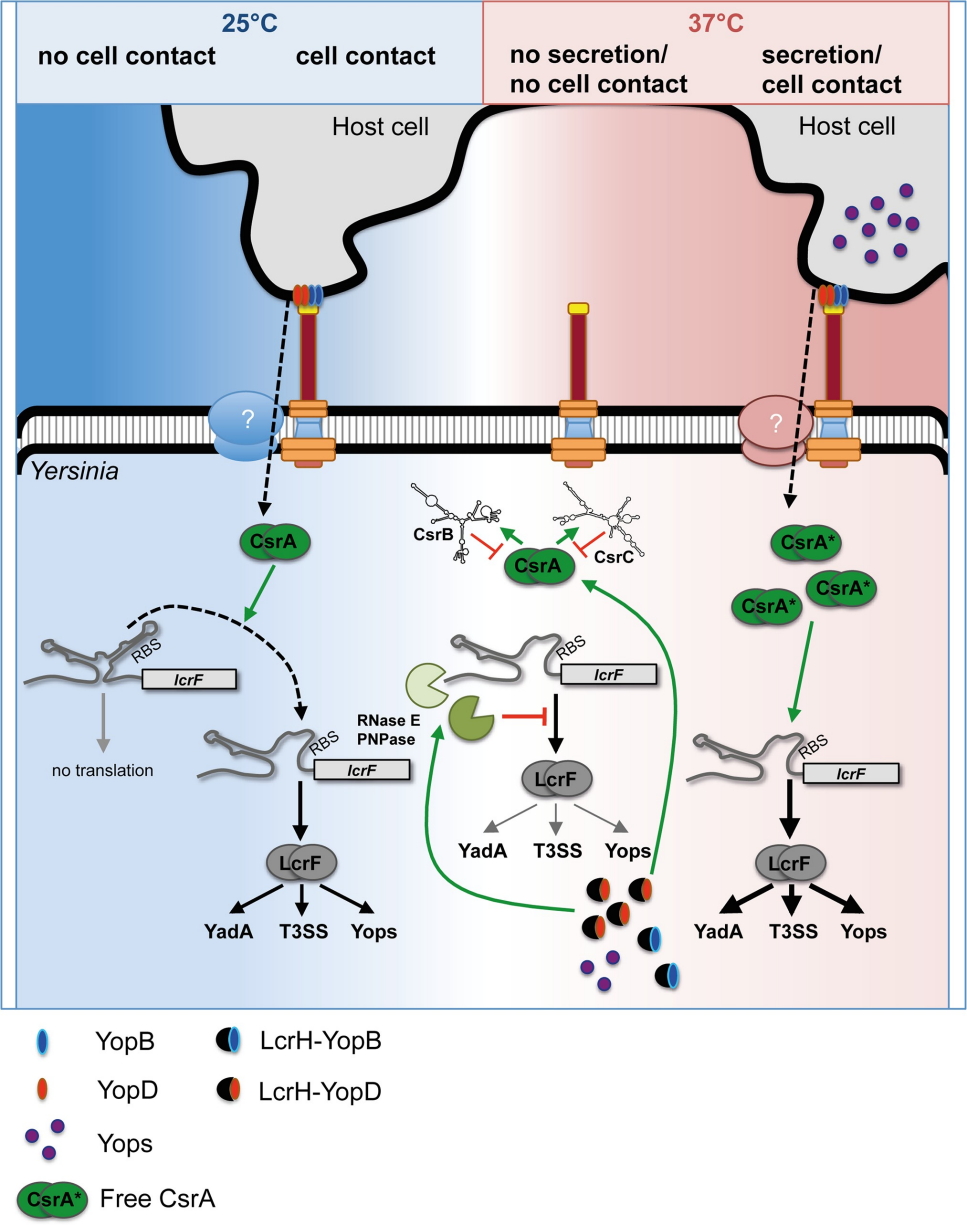
Model of CsrA and YopD-mediated control of LcrF synthesis in response to host cell contact. At 25°C, *lcrF* and *ysc/yop* gene expression is triggered by host cell contact. This requires the activity of CsrA to overcome translational repression by the LcrF RNA thermometer element. At 37°C, translational repression by the RNA thermometer is relieved and allows activation of *lcrF* translation and Ysc/Yop protein synthesis. In the absence of host cell contact, the Yop effectors bound to their chaperones accumulate in the bacterial cytoplasm. YopD represses LcrF expression by positive regulation of the degradosome components RNase E and PNPase, which promote *lcrF* transcript degradation. Moreover, presence of YopD influences the components of the Csr system in a way, that high levels of the antagonistic ncRNAs CsrB and CsrC are present that sequester and inhibit CsrA function. Reduced CsrA function prevents activation of *lcrF* translation and limits Ysc/Yop protein expression to a low level. Upon host cell contact, YopD is secreted and forms the translocation pore together with YopB, which leads to the depletion of YopD from the cytosol. This negatively affects the expression of RNase E and PNPase. Furthermore, loss of YopD triggers a change in the ratio of the Csr components in which significantly more CsrA is present in relation to CsrB and CsrC. The increased number of free and active CsrA molecules promotes a strong increase of LcrF and LcrF-mediated Ysc/Yop protein synthesis, which lead to the formation of active injectisomes and efficient secretion of the Yops into the host cell.

The underlying regulatory processes of the *Yersinia* plasmid-borne T3SS are innately complex. Physical contact between the pathogen and its target cell first triggers YopD secretion, which forms the translocon channel together with exported YopB. This event then induces effector translocation. Consequently, the intracellular concentration of YopD is lowered and, as shown in this study, this changes the levels of the components of the global carbon storage regulatory system (CsrABC) and the degradosome-associated RNases RNase E and PNPase. In this manner, T3S of YopD serves as a regulatory switch signaling host cell contact at body temperature. We found that YopD controls the synthesis of the global riboregulator CsrA directly and specifically in a post-transcriptional manner. Under non-secretion conditions, YopD interacts with sequences in the 5'-UTR of the *csrA* mRNA that overlap with a CsrA binding site located between promoter P2 and P1. Thereby, YopD interferes with the autoregulatory feedback circuit inhibiting CsrA translation from this transcript. The resulting modest reduction in the amount of CsrA upon YopD secretion is sufficient to destabilize and strongly reduce the overall amount of the antagonistic RNAs CsrB and CsrC, leading to a significant increase of the CsrA:CsrB/CsrC ratio and thus free functional CsrA (Fig 8). This is possible because CsrA, of which multiple molecules (≥8) interact with each sRNA, is absolutely essential for the maintenance of CsrB and CsrC, since it hinders CsrD to render them susceptible to RNase cleavage [36, 37]. As free CsrA boosts/intensifies translation initiation and enhances the stability of the *lcrF* transcript, we postulate that liberated CsrA from CsrB/C downregulation upon YopD secretion is responsible for the upregulation of LcrF levels in response to host cell contact (Fig 8). As many metabolic, physiological and stress adaptation/resistance functions, as well as important pathogenicity factors, are under control of the Csr system [30–33], we assume that an increase of CsrA activity upon host cell contact is used by the bacteria to rapidly remodel their overall fitness and virulence program to adjust to host cell responses.

Often proteins that repress translation work in concert with ribonucleases to accelerate the degradation of untranslatable mRNA [41]. Here we show, that this is also the case for the YopD-CsrA-T3SS/*yop* mRNA interplay: Presence of YopD reduces CsrA-mediated protection of *lcrF* mRNA decay and enhances the synthesis of *lcrF* mRNA degrading RNases RNase E and PNPase. PNPase is a phosphorolytic 3' to 5' exoribonuclease. It associates with RNase E, the RNA helicase RhlB, and enolase, forming the RNA degradosome and plays a major role in RNA turnover [42, 43]. RNase E-mediated cleavage often takes place within the 5' mRNA regions; this is followed by the processive exonuclease activity of PNPase to degrade target transcripts [41]. Notably, both ribonucleases were found to influence Yop production and secretion, but the precise molecular mechanism remained unclear [40, 43–45]. In this study, we found that *rne* and *pnp* transcript levels are reduced in the absence of YopD (*yopD* mutant or upon secretion). Since YopD interacts with the 5'-UTR of the *rne* and *pnp* transcripts, we hypothesize that YopD-binding increases the stability of the RNase transcripts. Although the molecular mechanism is still unclear, it is possible that YopD indirectly controls the expression of the RNase genes, e.g. in case of the *rne* gene. In addition, YopD could interfere with the negative autoregulation of RNase E and PNPase synthesis. For instance, YopD binding to the 5’-UTRs of the *pnp* transcript could hinder binding of both RNases, which cleave and process their own transcripts [46–48]. Alternatively, YopD could interfere with CsrA binding, which was also found to participate in the PNPase autoregulation in *E*. *coli* by repressing translation and reducing the stability of the *pnp* messenger [47, 49].

Although YopD binds specifically to 5' UTR sequences of the *csrA*, *pnp* and *rne* transcripts, the molecular mechanism how it recognizes and interacts with RNA is less clear. Work with *Y*. *enterocolitica* showed that YopD also directly interacts with multiple *yop* transcripts, prevents the recognition of translational initiation signals by ribosomes, and accelerates their degradation [22]. It was speculated that the specificity might be conferred by the presence of short linear AU-rich elements (AUAAA) in the YopD target mRNA near or overlapping the RBS. However, transfer of the AU-rich sequences to YopD-independent genes did not confer YopD control [22]. Moreover, sequences that are specifically recognized by YopD in the *csrA* 5'-UTR are not AU-rich. This observation and relative promiscuity of RNA binding *in vitro* at higher concentration, indicate that additional factors or sequence features of YopD target genes must exist. Furthermore, YopD interaction with the chaperone LcrH is necessary for repressing *ysc/yop* transcript translation [21, 50]. LcrH could be implicated in YopD-RNA binding or may only be required to hold YopD in an RNA-binding competent fashion.

Overall, YopD is one of the most abundant Yop proteins. This might ensure that even under non-secretion conditions still low intracellular levels of YopD are present for negative regulation. In parallel, the *lcrF* transcript is highly susceptible to degradation and particularly responsive to CsrA. CsrA directly activates LcrF synthesis on the post-transcriptional level by specific binding to sites overlapping with the RBS and nucleotides of codons 4–6 of the *lcrF* mRNA. Interaction of CsrA further stabilizes the *lcrF* transcript, most likely through the inhibition of RNase E/PNPase function and/or synthesis. Likewise, a deletion of the *csrA* homolog *rsmA* was recently shown to impair activation of the master regulator *hrpG* of the T3SS *hrp/hrc* genes in plant pathogenic *Xanthomonads* [51]. This indicates that this mechanism may be conserved among certain T3SS-encoding pathogens.

The model that emerges is that *Yersinia* T3SS/*yop* gene expression is multilayered and occurs in a sequential manner, beginning with a low-level induction of *lcrF* expression upon host entry, e.g. due to the rise of temperature. Formation of a translational block by YopD in the absence of host cell contact arrests T3SS/Yop synthesis on a low level and keeps expression control in a 'ready-to-go/standby' position. Flipping the switch, i.e. eliminating the blockage by secretion of YopD upon host cell contact triggers T3SS/Yop synthesis via modulation of the CsrABC-RNases cascade (Fig 7).

Intimate coupling of T3SS/effector gene transcription with cell contact-induced activation of the T3 secretory activity is common among T3SSs and was shown to involve complex regulatory networks and feedback control systems. However, the regulatory mechanisms involved are very diverse and differ significantly from *Yersinia*. In *Bordetella pertussis*, differential regulation of T3S genes is mediated by a secreted anti-sigma factor, which acts as an antagonist of an alternative sigma factor that controls the synthesis of T3SS apparatus components and secretion substrates [52]. Several other pathogens, including *Pseudomonas aeruginosa* and *Shigella flexneri* couple secretion and T3SS gene activation by a partner-switch mechanism [53–59]. This more widely used coupling theme implicates the transcriptional regulator of T3SS genes, a secreted protein, and additional interacting proteins. The latter change their binding partner when the secreted factor is injected into host cells and eliminated from the bacterial cytoplasm. This results in the liberation of the blocked transcriptional activator to activate T3SS gene transcription. Notably, the CsrA homolog RsmA of *Pseudomonas* was found to positively control T3SS gene expression in this microorganism, but the mechanism and whether this is linked to the partner-switching cascade is unclear [60, 61]. In enteropathogenic *E*. *coli*, the effector chaperone CesT, which is released and remains in the cytoplasm upon translocation of the effectors was shown to bind and antagonize CsrA. This modulates T3SS gene expression and known CsrA-repressed target genes [59].

The newly emerging regulatory scheme from our study now includes coupling cell contact-induced secretion of an RNA-regulating translocator protein with T3SS/effector gene expression through major global RNA regulators. This not only promotes a fast adaptation of T3SS/effector gene expression, it also coordinates T3SS with numerous CsrA- and RNase E/PNPase-dependent virulence-relevant traits, stress adaptation and metabolic functions in response to host cell contact. This reprogramming is crucial to ensure adaptation, survival, proliferation, and pathogenesis during acute infections. In particular, it not only prevents sudden immune cell attacks, but also adjusts the overall fitness of the pathogen towards the imposed energetic burden entailed by fully active T3SSs. Moreover, the RNA-based regulatory circuit can also trigger down-regulation of T3SS/Yops and other virulence factors during later stages of the infection, when immune cell attack has been largely defeated, and thereby contribute to the initiation of persistent infections [62, 63]. We postulate that T3SS activity of *Yersinia* provokes a global remodeling of the virulence and fitness program through control of CsrA and the RNA degradosome by YopD, which is crucial for the pathogens to adapt to rapidly changing conditions and flourish in different hostile host niches. As the CsrA/RsmA regulator was found to control the expression of many T3SSs genes in Gram-negative bacteria [32], we propose that implementing CsrA/RsmA as an interaction partner of T3SS-secreted proteins herein delineates a general strategy for responding to host cell contact. The challenge ahead lies in unifying all identified interactions and mutant phenotypes in the different pathogens into a regulatory network explaining previous and future observations.

## Material and methods

### Bacterial strains, cell culture, media and growth conditions

*E*. *coli* and *Yersinia* strains were routinely grown under aerobic conditions at 25°C or 37°C in LB (Luria Bertani) broth on solid or in liquid media if not indicated otherwise. The antibiotics used for bacterial selection were as follows: carbenicillin 100 μg ml^-1^, chloramphenicol 30 μg ml^-1^, tetracycline 10 μg ml^-1^, and kanamycin 50 μg ml^-1^. For northern blot experiments bacteria were diluted 1/50 in fresh medium from overnight cultures, grown to exponential phase (OD_600_ 0.5) at 25°C and then shifted to 37°C for additional 4 h in the absence (+Ca^2+^, T3SS/*yop* gene non-inducing conditions) or presence of 20 mM MgCl_2_ and 20 mM sodium oxalate to deplete Ca^2+^ ions (-Ca^2+^, T3SS/*yop*-inducing conditions). HEp-2 cells (ATCC, USA) used for the analysis of host cell contact expression assays were grown in a humidified atmosphere with 5% CO_2_ at 37°C (HERA cell 150 incubator, Thermo Scientific) in RPMI1640 supplemented with Glutamax medium containing 7.5% NCS as growth factor.

### DNA manipulation, construction of plasmids and mutant strains

All DNA manipulations, restriction digestions, ligations and transformations were performed using standard genetic and molecular techniques [64]. The plasmids used in this work are listed in S1 Table. Oligonucleotides used for PCR, qRT-PCR and sequencing were purchased from Metabion and are listed in S2 Table. Plasmid DNA was isolated using Qiagen plasmid preparation kits. DNA-modifying enzymes and restriction enzymes were purchased from New England Biolabs, Promega and Roche. PCRs were performed in a 100 μl mix for 29 cycles using Phusion High-Fidelity DNA polymerase (New England Biolabs). Purification of PCR products was routinely performed using the QIAquick PCR purification kit (Qiagen). The constructed plasmids were sequenced by the in-house facility.

Plasmid pAKH172 is derived from pET28a, carrying a His_6_-tag. The *csrA* coding region was amplified by PCR with primers IV783/I68. The resulting insert was digested with *Nco*I and *Xho*I and ligated into the *Nco*I/*Xho*I site of pET28a. To construct pJE9, the *yadA* insert was amplified via PCR with primers 90/92. The resulting fragment was digested with *Bam*HI and *Kpn*I and ligated into the *Bam*HI/*Kpn*I site of pGFPmut3.1 to produce pJE2. Next, pJE2 was digested with *Pst*I and *Spe*I, and the resulting *yadA*-*gfp* fragment was ligated into the *Pst*I/*Spe*I site of pIV2mob. The kanamycin cassette from pJE9 was exchanged against the ampicillin cassette. To do so, the *bla* gene was amplified by PCR with primers I972 and I998 using pBADmycC as template. The resulting fragment was digested with *Bgl*II and ligated into the *Bgl*II site of pJE9. The *csrA-lacZ* reporter gene fusion plasmids pJH4 and pJH6 were constructed by amplification of the *csrA* upstream region using primer pairs IV436/II275 and IV438/II275. After digest with *Eco*RI and *Pst*I, the inserts were ligated into the *Eco*RI/*Pst*I site of pKB63. To construct pJH12 the *yopD* gene was amplified with primers IV706/III645 and the *lcrH* gene with primer pair IV708/III647. Next, the *yopD*, as well as the *lcrH* inserts, were digested with *Nde*I/*Spe*I and *Spe*I/*Xho*I, respectively. Subsequently, the fragments were ligated into the *Nde*I/*Xho*I sites of linearized pET28a. For construction of the low copy plasmid pKB60, the *csrA* coding region was amplified with the primer pair 558/559. The resulting fragment was digested with *Sal*I and *Bam*HI and ligated into the *Sal*I/*Bam*HI sites of pHSG576. Plasmid pKB99 was derived from pED7 carrying the *lcrF-lacZ* fusion with a mutation which destabilizes the thermoloop structure in the 5’-UTR of *lcrF* (GUU -30/-28 AAA). The kanamycin cassette was synthesized with primers III902 and III905, digested with *Xba*I and *Sph*I and ligated into the *Xba*I/*Sph*I sites of pED07. Plasmid pMP1 was derived from pBAD33. The *lcrF gene* fragment (-123 to +771 relative to the translational start of *lcrF*) was amplified by PCR using primer pairs V659/I214. The produced fragment was digested with *Xba*I and *Sac*I and ligated into the *Xba*I/*Sac*I sites of pBAD33. For construction of the plasmid pFU100, the Ap^R^ cassette of pFU72 was exchanged against the Cm^R^ cassette of pZA31-luc. Therefore, pZA31-luc was digested with *Xho*I and *Sac*I, and the isolated fragment was ligated into the *Xho*I/*Sac*I sites of pFU72. The plasmid pRS1 was constructed from pFU50. The kanamycin cassette of pFU50 was excised with *Sac*I and *Xho*I and replaced by the chloramphenicol cassette of pZA31*luc*. The translational *rne-lacZ* and *pnp-lacZ* fusions of pIVO22 and pIVO23 were constructed by PCR amplification of the *rne* and *pnp* promoter fragments using primer pairs VIII675/VIII677 and VIII672/VIII674, which were ligated into the *Bam*HI/*Sal*I site of pTS02. The corresponding transcriptional *rne-lacZ* and *pnp-lacZ* fusions of pIVO25 and pIVO26 were constructed by amplification of the *rne* and *pnp* promoter fragments using primer pairs VIII675/VIII676 and VIII672/VIII673, which were ligated into the *Bam*HI/*Sal*I site of pTS03. To construct pRS2, the *yopD* gene was amplified by PCR with the primer pair II348/II349. The resulting fragment was inserted into the *Pst*I and *Not*I sites of pRS1. The *lcrQ* fragment was produced with primers II360 and II361 and ligated into the *Pst*I/*Not*I sites of pRS1 generating pRS4. To construct pRS18, the midi copy origin p15A was amplified by PCR with primers II496/II538 using pACYC184 as template. The insert was digested with *Sac*I and *Xba*I and introduced into the *Sac*I and *Xba*I sites of pRS4. For construction of pRS15, the *lcrQ* coding region of pRS18 was removed by digestion of the vector with *Mlu*I and *Sph*I. The sticky ends of both restriction sites were blunted and the vector was religated using the CloneJET PCR Cloning Kit (Fermentas, USA). Plasmid pRS16 was generated by exchanging the ori29807 of pRS2 with the p15A origin as described for pRS18. To construct pRS40, an *rne* fragment (-15 to +1395 nt with respect to the *rne* start codon) was amplified by PCR with the primer pair III233/III234 and cloned into the *Xba*I and *Sph*I sites of pBAD33. For the construction of pRS50, the kanamycin cassette was amplified with the primer pair I661/I662 using pKD4 as template. Next, about 500 bp of the upstream region of *pnp* was amplified with the primer pair III243/III244. Primer III244 possesses additional 20 nt at the 5´-end that are homologous to the start of the kanamycin resistance gene. The *pnp* downstream fragment was amplified with primer pair III245/III246. The forward primer III245 contained additional 20 nt at the 5´-end that were homologous to the end of the kanamycin resistance gene. Next, a PCR reaction was performed with primer pair III243/III246 using the upstream and downstream PCR products of the *pnp* and the kanamycin cassette as templates. Finally, the PCR product was digested with *Sac*I and integrated into the *Sac*I site of the suicide vector pAKH3. The mutagenesis plasmid pRS34 (Δ*yopD*) was constructed by amplification of 300 bp regions upstream (II363/II366) and downstream (II364/II365) of the *yopD* coding sequence. The reverse (II366) and the forward (II365) primers contain overlapping sequences complementary to each other at their ends. Another PCR, using primers II365/II364 and the two fragments as template, resulted in one fragment with an in-frame deletion of the *yopD* gene. The fragment was digested with *Spe*I and *Sph*I, and integrated into the *Spe*I/*Sph*I site of the suicide plasmid pDM4 [65]. For the construction of pSR1, the *gfp*_*mut3*.*1*_ gene was excised of pGFPmut3.1 with *Pst*I and *Spe*I and ligated into the *Pst*I/*Spe*I sites of pIV2mob. The *yscW-lcrF* locus was excised from pWO8 with *Bam*HI and *Sa*lI and ligated into the BamHI/*Sa*lI sites of pFU98 generating pTS34. To construct pWO3, an insert carrying P_*yscW*_, the *yscW* deletion (+113 to +298 nt with respect to the *yscW* transcriptional start site) as well as the first 72 nucleotides of the *lcrF* coding sequence was synthesized via a three-step PCR with primers I222/I224 using two fragments amplified from *Yersinia* genomic DNA with the primer pair I224/I747 and I222/I746, respectively, as template. This insert was digested with *Pst*I and ligated into the *Pst*I site of pGP20, resulting in pKB12. Next, the P_*yscW*_::Δ*yscW-lcrF’* insert was amplified with the primer pair I224/I964 using pKB12 as template, digested with *Pst*I and *Bam*HI, and finally ligated into the *Ps*tI/*Bam*HI sites of pSR1. To generate pWO8, the *yscW-lcrF* locus was amplified with primers I214/I844. The insert was digested with *Xba*I and *Sph*I and ligated into pACYC184 resulting in pKB28. Next, the *yscW-lcrF* fragment was extracted from pKB28 and cloned into pFU51 via the *Sal*I/*Xba*I restriction sites. Plasmids pWO41 (*yadA*-*luxCDABE*) and pWO42 (*lcrF*-*luxCDABE*) were constructed by an exchange of the chloramphenicol resistance gene of pTS31 and pTS34 against the ampicillin resistance gene of pFU31 using the *Sac*I/*Bam*HI sites. For the construction of pWO14, the pSC101* ori of pWO8 was exchanged against the suicide ori R6KmobRP4 of pFU100 via the *Sac*I/*Xba*I restriction sites generating pWO13. Next, the non-coding region between YPTB1128 and YPTB1129 was amplified by PCR with primers II341/II342 and integrated into the *Sac*I restriction site of pWO13.

### Construction of *Y*. *pseudotuberculosis* deletion mutants

To generate YP155, plasmid pWO14 was mated from *E*. *coli* S17-1 λpir (*tra*^+^) into *Y*. *pseudotuberculosis* YP12 and transconjugants were selected on *Yersinia* selective agar (Oxoid) supplemented with carbenicillin. All other mutant strains with integrations, deletions, resistance cassette insertions or nucleotide substitutions were constructed by homologous recombination using suicide plasmids pRS34 (YP91, YP145) and pRS50 (YP138). Plasmids were mated from *E*. *coli* S17-1 λpir (*tra*^+^) into *Y*. *pseudotuberculosis* YPIII and transconjugants were selected on *Yersinia* selective agar (Oxoid) supplemented with chloramphenicol. The recombination of the plasmid into the *Yersinia* virulence plasmid pYV yielded a merodiploid strain, including a wildtype and the mutant copy. Subsequently, the resulting strain was plated on 10% sucrose to induce expression of the toxic *sacB* gene on the suicide plasmids. 50 selected fast-growing strains were screened for chloramphenicol sensitivity to prove the loss of the integrated plasmid. All mutant strains were proven by PCR and DNA sequencing.

### RNA isolation, northern blotting and RNA degradation assays

Bacteria grown under the required growth conditions were pelleted and RNA was isolated using the SV total RNA purification kit (Promega) as described [34]. Total RNA (20 μg) was separated on MOPS agarose gels (1.2%), transferred by vacuum blotting for 1.5 h onto positively charged membranes (Whatman) in 10 x SSC buffer (1.5 M NaCl, 0.15 M sodium citrate, pH7) using a semi-dry blotting system and UV cross-linked. Prehybridization, hybridization to DIG-labelled probes and membrane washing were conducted using the DIG Luminescent Detection Kit (Roche, Germany) according to the manufacturer's instructions. The DIG-labelled PCR fragments used as probes were produced by PCR using the DIG-PCR nucleotide mix (Roche, Germany) as described [34] with the following primer pairs: for the *lcrF* transcript—I214/I303, for the *csrB* and *csrC* transcripts—555/556 and 583/I82, for the *rne* transcript—IV529/IV530 and the *pnp* transcript—IV527/IV528 (see S2 Table).

To determine stability of the *lcrF* transcript, RNA stability assays were performed. In order to stop the *de novo* mRNA synthesis 0.5 mg/ml rifampicin (Serva) was added. 0, 1, 2, 3, 5 and 7.5 min after rifampicin treatment, 10% v/v phenol was added and the samples were snap frozen in liquid nitrogen. RNA isolation and northern blot analysis were performed as described above.

### Quantitative real-time RT-PCR (qRT-PCR)

qRT-PCR was performed using the SensiFastNoRox Kit (Bioline) with 25 ng/μl of the RNA samples according to the manufacturer's instructions. qRT-PCR was performed in a Rotor-Gene Q lightcycler (Qiagen). Primers used for analyzing relative gene expression purchased from Metabion and are listed in S2 Table. The gene *sopB* was used for normalization. Data analysis was performed with the Rotor-Gene Q Series Software. Relative gene expression was calculated as described earlier [66]. Primer efficiencies were determined experimentally using serial dilutions of genomic *Y*. *pseudotuberculosis* YPIII DNA. Primer efficiencies are: *csrB*: 2; *csrC*: 2; *pnp*: 2; *rne*: 2; *sopB*: 2.

### Purification of the CsrA protein and the YopD-LcrH complex

*E*. *coli* strain BL21 transformed with pAKH172 or pJH12 was grown at 37°C in LB broth to an A_600_ of 0.6. 0.5 mM IPTG was used to induce CsrA-His_6_ and YopD-His_6_ production. CsrA-His_6_ purification was performed as described previously [67]. The His_6_-YopD protein was purified by Ni-NTA affinity chromatography in lysis buffer (50 mM Na_2_HPO_4_/NaH_2_PO_4_, pH 8.0, 300 mM NaCl, 20 mM imidazole). The column was washed two times with three column bed volumes of lysis buffer supplemented with 40 mM imidazole. Bound YopD was eluted by adding six column bed volumes of lysis buffer supplemented with 250 mM imidazole. For RNA electrophoretic mobility shift assays, the proteins were dialyzed over night against the respective RNA-binding buffer (see RNA-EMSA) at 4°C. The purity of CsrA-His_6_ and the YopD-His_6_-LcrH complex was estimated to be >95%.

### *In vitro* transcription of *csrA*, *lcrF*, *rne* and *pnp* transcripts

The *csrA*, *lcrF*, *rne* and *pnp* transcripts for the RNA electrophoretic mobility shift experiments were obtained by run-off transcription with T7 RNA polymerase from PCR fragments. The double-stranded DNA templates were amplified from chromosomal DNA of the *Y*. *pseudotuberculosis* wildtype strain YPIII with specific primer pairs for the region, of which the forward primer contained a T7 promoter sequence (*lcrF*: -123 to +15 with V731/V732 and -123 to +75 with V731/I404, *csrA*: -91 to +10 with V066/III731, *csrA*: -91 to -40 with V066/V631, *csrA*: -105 to -60 with V830/V831, *csrA*: -42 to +10 with V708/III731, *csrA*: -50 to -+10 with VI386/III731, *csrA*: -70 to -+10 with V832/III731, *hns*: +175 to +226 V700/I515, *rne*: -148 to +12 VI564/VI565, *pnp* -141 to +12 VI950/VI085, see S2 Table). PCR reaction was performed with the Phusion-HF polymerase (NEB, USA). The *csrA* fragment -105 to +10 (Δ -70 to -40) and the *lcrF* fragment -123 to +75 harboring the GGA to TTC exchange were ordered as gblocks gene fragment from Integrated DNA Technologies. The PCR fragments were purified using the NucleoSpin Gel and PCR Clean-up Kit (Macherey-Nagel, Germany). The fragments were transcribed *in vitro* using the TransAid T7 High Yield Transcription Kit (Thermo Scientific, USA) at 37°C for 2 h according to the manufacturer's recommendation. Template DNA was digested with DNase I for 15 min at 37°C, and the enzyme inactivated at 65°C for 10 min. Finally, the RNA run-off transcript was purified by phenol-chloroform extraction [64].

### RNA electrophoretic mobility shift assay (RNA-EMSA)

For the RNA-EMSAs, some RNAs were 3’-end labeled with pCp Biotin (Jena, Bioscience, Germany) by ligation with T4 RNA Ligase I (*lcrF* transcripts, Fig 2A; *csrA* (a) transcript, Fig 5B and 5C; *csrA* transcript (e) and (f), Fig 5C). The reaction was performed with 1 μg linearized RNA in 1 x T4 ligase buffer, 1 mM ATP, 10% (w/v) DMSO, 1 μM pCp-Biotin (Jena, Bioscience, Germany), 15% (w/v) PEG8000 and 1 μl T4 ssRNA ligase (20 u, NEB, USA) at 18°C for 2 h or overnight. The labeled RNA fragments were purified by phenol-chloroform extraction [64], and RNA labeling was checked by northern blotting. To do so, 2 nM RNA were separated on a native 4–8% TBE polyacrylamide gel, transferred onto a nylon membrane with a Trans-Blot SD Semi-Dry Transfer Cell (Biorad, Germany) and crosslinked to the membrane with an UV-crosslinker (Stratagene, USA). The biotinylated RNAs were detected with the Chemiluminescent Nucleic Acid Detection Module (Thermo Scientific), and light emission was detected by a ChemiDoc XRS+ (Biorad, USA). All other transcripts used for the RNA-EMSAs were 5’-end labelled with radioactive P^32^ γ-ATP (10 μCi/μl) using the polynucleotide kinase (Thermo Fisher Scientific). The reaction was performed with 100 ng linearized RNA in 1 x polynucleotide kinase buffer, and 1 mM P^32^ γ-ATP at 37°C for 1 h. The reaction was stopped with STE buffer. The labeled RNA fragments were purified by phenol-chloroform-isoamyl alcohol extraction [64], and RNA labeling was checked by northern blotting.

For RNA-binding studies the purified CsrA and YopD proteins were dialyzed against the RNA-binding buffer (20 mM Na_2_HPO_4_/NaH_2_PO_4_ (pH 8,0), 100 mM KCl, 5% (w/v) glycerol, 2 mM DTT). The different biotinylated *lcrF*, *csrA*, *rne* and *pnp* transcripts used for RNA band shift analysis were diluted in the equivalent RNA-binding buffer, denatured at 70°C for 10 min and chilled on ice. Subsequently, the transcripts (20 fmol/2 nM RNA) were incubated with increasing concentrations of purified CsrA or YopD for 30 min on ice in the RNA-binding buffer, and immediately loaded on 4–8% polyacrylamide gels. After electrophoresis the RNA and RNA-protein complexes were transferred on a nylon membrane and visualized as described above.

### *In vitro* translation of *csrA*

To test the influence of CsrA on *lcrF* mRNA translation, an *in vitro* translation assay was performed using PURExpress *In vitro* protein Synthesis Kit (NEB, USA) according to the manufacturer's instructions. *In vitro* transcribed *lcrF* RNA from plasmid pMP1 was used as template.

### Gel electrophoresis and western blotting

For immunological detection of CsrA, YopD, YadA, LcrF and the Yop proteins, cell extracts of equal amounts of bacteria were prepared and separated on a 15% polyacrylamide SDS gel [64]. Proteins were transferred onto an Immobilon-P membrane (Millipore) and probed with polyclonal antibodies directed against CsrA, YopD, LcrF, YadA or secreted Yop proteins (Davids Biotechnologies, Germany) as described [67].

### Host cell contact assays

Host cell contact-dependent expression of *Yersinia* virulence genes was assessed with *Y*. *pseudotuberculosis* strain YPIII and isogenic mutant strains (YP53 Δ*csrA*) with or without contact to HEp-2 cells (ATCC, USA) by a) *in situ* monitoring of *gfp* reporter fusions by fluorescence microscopy, b) *in situ* quantification of light emission of *luxCDABE* reporter fusions and, and c) detection and quantification of LcrF, YadA or YopE.

For the microscopical monitoring *Y*. *pseudotuberculosis* strains expressing the respective *gfp* reporter fusions were grown at 25°C to stationary growth phase. About 10^6^ HEp-2 cells seeded in μ-slide 8-well microscope slides (ibidi, Germany) were infected with about 5 x 10^7^ bacteria. The bacteria were centrifuged onto the epithelial cells and incubated up to four hours at 25°C or 37°C. Fluorescence emitted by GFP was analyzed by fluorescence microscopy (Axiovert II with Axiocam HR, Zeiss, Germany) using the AxioVision program (Zeiss, Germany).

For the monitoring of bioluminescent *luxCDABE* reporter fusions *Y*. *pseudotuberculosis* strains harboring promoter fusions to the bacterial luciferase operon *luxCDABE* were grown at 25°C to stationary growth phase. 5 x 10^4^ HEp-2 cells seeded in 96 well assay plates (Corning Incorporated) were infected with 10^6^ bacteria. The bacteria were centrifugated onto the cells and incubated for 2.5 h at 25°C in a Varioscan plate reader (ThermoScientific). Light emission was measured every 10 min and documented with the Varioscan Flash and SkanIt RE software (ThermoScientific). Curves of relative luminescent units (RLU) per time were calculated out of the triplicates, normalized to the starting conditions and visualized by GraphPad Prism.

To visualize a host cell contact-mediated increase of the YadA and the LcrF transcript and protein levels in the different tested *Y*. *pseudotuberculosis* strains, 2 x 10^8^ bacteria were used to infect 4 x 10^6^ HEp-2 cells. The bacteria were centrifuged onto the cells and incubated for 150 min at 25°C or 37°C. The infected cell culture was washed with PBS, lysed (0.1% Triton X-100, 0.9% NaCl), and diluted in stop solution for RNA isolation and northern blotting, or in SDS sample buffer for SDS-PAGE and western blotting.

### Yop secretion assay

For Yop secretion assays, about 50 ml LB medium were inoculated 1:50 with an overnight culture of the desired strain and grown at 25°C for 2 h. Subsequently, secretion was induced by addition of 20 mM MgCl_2_ and 20 mM sodium oxalate. The cultures were shifted to 37°C and cultivated for 4 h. Cell quantities of the different bacterial cultures were adjusted according to their OD_600_. The bacteria were harvested in falcon tubes by centrifugation. The supernatant was filtered and proteins were precipitated with 1/10 volume of 100% trichloroacetic acid (TCA). After incubation for 20 min on ice, the proteins were pelleted. The pellets were resuspended in 2 ml acetone-SDS solution (1.75 ml 100% acetone, 0.25 ml 2% SDS), incubated on ice for 20 min, and pelleted again. To wash the proteins, the supernatant was discarded and 500 μl 100% acetone was applied. After the last centrifugation step, the precipitated proteins were resuspended in equal amounts of sample buffer and applied on 15% SDS polyacrylamide gels for electrophoretic separation. Coomassie Brilliant Blue was used to visualize the proteins.

### Luciferase and β-galactosidase assays

Bacteria harboring *luxCDABE* and *lacZ* reporter fusion plasmids were grown under different growth conditions as described. β-galactosidase was measured in cell-free extracts as described previously [68]. The activities were calculated as follows: β-galactosidase activity OD_420_·6,75·OD_600_^-1^·Δt (min)^-1^·Vol (ml)^-1^ β-galactosidase assays were performed in triplicate of cultures grown under indicated conditions. Reporter fusions emitting bioluminescence were measured in non-permeabilized cells with a Varioscan Flash (Thermo Scientific) using the SkanIt software (Thermo Scientific) for 1 s per time point and every 10–15 min for kinetic analyses. The data are given as relative light units (RLU/OD_600_) from three independent cultures performed in duplicate.

## Supporting information

S1 Table Bacterial strains and plasmids. The table includes all bacterial strains and plasmids used in this study.
(DOCX)

S2 Table Oligonucleotides. The table includes all oligonucleatides used in this study.
(DOCX)

S1 References References for S1 Table. (DOCX)

S1 Fig Cell contact-induced expression of YadA and LcrF. (A) Left panel: extracts of strain YPIII (WT) resuspended in the cell culture supernatant (SN) and extracts of uninfected HEp-2 cells. Middle panel: strain YPIII (WT) incubated for 2 h at 25°C with (right) or without (left) seeded HEp-2 cells. Right panel: strain YPIII (WT) and the isogenic virulence plasmid-cured strain (pYV^-^) grown in LB under secretion conditions (37°C, in the absence of Ca^2+^). After 2 h HEp-2 cells were lysed with 0.1% Triton, whole cell extracts were prepared and YadA and LcrF were detected by western blotting using polyclonal antibodies directed against YadA and LcrF. Polyclonal anti-H-NS antibodies were used to detect H-NS, which served as loading control. Furthermore, whole cell extracts of the virulence plasmid-cured strain YP12 (pYV^-^) and the wildtype strain YPIII (WT) grown under secretion conditions at 37°C in the absence of Ca^2+^ (right panel) were used as negative and positive controls. (B) YPIII (WT) strains harboring *lcrF-luxCDABE* (pTS34), *yadA-luxCDABE* (pTS31), *invA-luxCDABE* (pTS32), or *gapA-luxCDABE* (pFU166) fusion plasmids, were used to infect HEp-2 cells in PBS or incubated in PBS without cells at 25°C. Bioluminescence of the samples was monitored after 2 h. The data represent the mean ± SD of the fold change (end/start) from three independent biological replicates and were analyzed with Student’s t-test: ***: P<0,001, n.s. (P>0.05). (C) HEp-2 cells infected with *Y*. *pseudotuberculosis* wildtype strain YPIII harboring pJE2 (empty vector) or a plasmid-encoded *yadA-gfp* (pJE9) and Δ*lcrF* strain carrying plasmid-encoded *yadA-gfp* (pJE9) incubated for 4 h at 25°C. White bars indicate 5 μm.
(TIF)

S2 Fig CsrA- and YopD-dependent, host cell contact-mediated induction of YadA production. (A) HEp-2 cells were infected with YPIII (WT), YP53 (Δ*csrA*) and YP53 pAKH56 (Δ*csrA*) p*csrA*^*+*^ at 25°C (+). After 2 h the cells were lysed with 0.1% Triton and samples for SDS-polyacrylamide electrophoresis and western blot analysis with polyclonal anti-YadA or anti-H-NS (control) sera were prepared. YadA expression was visualized in bacteria incubated in PBS without cells (-) or with HEp-2 cells (+) or in bacteria that were used to start HEp-2 infection (st). (B) HEp-2 cells were infected with YPIII (WT), YP53 (Δ*csrA*), YP91 (Δ*yopD*), and YP145 (Δ*csrA*, *yopD*) at 25°C (+). After 2 h samples for SDS-polyacrylamide electrophoresis and western blot analysis with polyclonal anti-YadA and anti-H-NS (control) sera were prepared. YadA expression was visualized in bacteria incubated in PBS without cells (-) or with HEp-2 cells (+) or in bacteria that were used to start HEp-2 infection (st).
(TIF)

S3 Fig Analysis of CsrA and CsrC levels under different T3SS-manipulating conditions. *Y*. *pseudotuberculosis* strain YPIII (WT) pV (empty vector pRS1), YP91 (Δ*yopD*) pV (empty vector pRS1), YP91 (Δ*yopD*) p*yopD*^+^ (pRS2), YP53 (Δ*csrA*) pV (empty vector pRS1), YP53 (Δ*csrA*) p*csrA*^+^ (pKB60), YP53 (Δ*csrA*) p*yopD*^+^ (pRS2) were used to analyze reciprocal complementation of YopD and CsrA of a *csrA* and *yopD* mutant by monitoring CsrC transcript levels. The *csrB*-deficient strain YP52 was used as negative control. Total RNA of the cultures was prepared and subjected to northern blotting using a CsrC-specific probe. The 16S and 23S rRNAs served as loading controls. A representative of at least three independent experiments is presented.
(TIF)

S4 Fig Influence of YopD on the expression of transcriptional and translational *rne-lacZ* and *pnp-lacZ* fusions. Strains YPIII (WT) pRS15 (empty vector), the YP91 (Δ*yopD*) pRS15 (empty vector), and YP91 (Δ*yopD*) pRS16 (*yopD*^+^) harboring transcriptional or translational *rne-lacZ* or *pnp-lacZ* reporter plasmids were grown to late exponential phase at 37°C. β-galactosidase activity of the different cultures was monitored. The data represent the mean ± SD from at least three independent biological replicates performed in triplicates and were analyzed with Student’s t-test; ****: P<0,0001.
(TIF)
