# Supplementary material for: A bacterial secreted translocator hijacks riboregulators to control type III secretion in response to host cell contact
Source: PLoS Pathog. 2019 Jun 7;15(6):e1007813. doi: 10.1371/journal.ppat.1007813 (PMC6583979; doi:10.1371/journal.ppat.1007813)
Supplement: S1 Table — The table includes all bacterial strains and plasmids used in this study. (DOCX) [file ppat.1007813.s001.docx]

**Table S1: Bacterial strains and Plasmids**

| **Strains/**  **Plasmids** | **Description** | **Source and Reference** |
| --- | --- | --- |
| **Strains** |  |  |
| *E. coli* |  |  |
| CC118 | F^-^ Δ(*ara-leu*) 7679 Δ(*lacZ*) 74Δ(*phoA*)20 *araD139 galE galK thi rpsE rpoB arfE^am^ recA1,* *λpir* | [[1](#_ENREF_69)] |
| S17-1λpir | *recA1 thi pro hsdR^-^* RP4-2Tc::Mu Km::Tn7 λpir | [[2](#_ENREF_70)] |
| BL21λDE3 | F- *ompT gal dcm lon hsdSB (rB2 mB2) gal* λDE3 | [[3](#_ENREF_71)] |
|  |  |  |
| *Y. pseudo­tuberculosis* |  |  |
| YPIII | + pYV, wildtype | [[4](#_ENREF_72)] |
| YP12 | - pYV, wildtype strain cured of the virulence plasmid | [[5](#_ENREF_73)] |
| YP52 | YPIII Δ*csrC,* Kn^R^, Δ*csrB*, Ap^R^ | [[6](#_ENREF_67)] |
| YP53 | YPIII Δ*csrA*, Kn^R^ | [[6](#_ENREF_67)] |
| YP66 | YPIII Δ*lcrF*, Ap^R^ | [[7](#_ENREF_34)] |
| YP91 | YPIII Δ*yopD* | this study |
| YP138 | YPIII Δ*pnp* | this study |
| YP145 | YPIII Δ*yopD,* Δ*csrA,* Kn^R^ | this study |
| YP155 | YPIII - pYV, *lcrF*^+^, Ap^R^ | this study |
|  |  |  |
| **Plasmids** |  |  |
| pACYC184 | cloning vector, ori p15A, Tet^R^, Cm^R^ | [[8](#_ENREF_74)] |
| pAKH3 | pGP704, *sacB*^+^, Ap^R^ | [[9](#_ENREF_29)] |
| pAKH56 | pACYC184, P*_csrA_-csrA*^+^, p15A, Cm^R^ | [[6](#_ENREF_67)] |
| pAKH172 | pET28a, *csrA*^+^, Kn^R^ | this study |
| pBAD18-*lacZ*(481) | translational *lacZ* fusion vector, Ap^R^, ori pBR, ori M13 | [[10](#_ENREF_75)] |
| pBAD33 | expression vector, P*_BAD_*, *araO2*, *araC*, ori p15A, Cm^R^ | [[11](#_ENREF_76)] |
| pBADmycC | expression vector, N-terminal His_6_-tag, P*_BAD_*, ori pBR322, Ap^R^ | Invitrogen |
| pDM4 | mutagenesis vector, ori R6K, *sacB*, Cm^R^ | [[12](#_ENREF_65), [13](#_ENREF_77)] |
| pED07 | pBAD18-*lacZ*(481), pBAD::*lcrF-lacZ* (-124 to +74)^a^, mutation GUU-30/-28AAA), Ap^R^ | [[7](#_ENREF_34)] |
| pET28a | T7 overexpression vector, ori 3286, Kn^R^ | Novagen |
| pFU31 | *gfp_mut3.1_*, ori ColE1, Ap^R^ | [[14](#_ENREF_27)] |
| pFU50 | *dsRed2*, P*_lac_*, ori 29807, Kn^R^ | [[14](#_ENREF_27)] |
| pFU51 | *gfp-*RBS*,* pSC101*, Ap^R^ | [[14](#_ENREF_27)] |
| pFU72 | *luxCDABE*, R6K mobRP4, Ap^R^ | [[14](#_ENREF_27)] |
| pFU98 | promoterless *luxCDABE* operon, ori pSC101*, Cm^R^ | [[14](#_ENREF_27)] |
| pFU100 | *luxCDABE*-RBS, R6Kmob, Cm^R^ | this study |
| pFU166 | *gapA-luxCDABE,* ori colE1, Ap^R^ | [[14](#_ENREF_27)] |
| pGFPmut3.1 | *gfp_mut3.1_* expression vector, ori pUC, Ap^R^ | Clontech |
| pGP20 | promoterless *lacZ* gene, ori pSC101, Tet^R^ | [[7](#_ENREF_34)] |
| pHSG576 | cloning vector, ori pSC101, Cm^R^ | [[15](#_ENREF_78)] |
| pIV2mob | *Yersinia* cloning vector, ori 29807, Ap^R^ | [[13](#_ENREF_77)] |
| pIVO22 | pT02, *rne-lacZ* (-646 to +44)*,* ori pSC101, Ap^R^ | this study |
| pIVO23 | pT02, *pnp-lacZ* (-288 to +44)*,* ori pSC101, Ap^R^ | this study |
| pIVO25 | pT03, *rne-lacZ* (-646 to -315)*,* ori pSC101, Ap^R^ | this study |
| pIVO26 | pT03, *pnp-lacZ* (-288 to -87)*,* ori pSC101, Ap^R^ | this study |
| pJE2 | pGFPmut3.1, *yadA-gfp*, ori pUC, Ap^R^ | this study |
| pJE9 | pJE2, *yadA-gfp,* ori p29807, Kn^R^ | this study |
| pJH4 | pKB63, *csrA’-’lacZ* (-298 to +17)^c^, ori pSC101, Ap^R^ | this study |
| pJH6 | pKB63, *csrA’-’lacZ* (-121 to +17)^c^, ori pSC101, Ap^R^ | this study |
| pJH12 | pET28a, *yopD*^+^, *lcrH*^+^, Kn^R^ | this study |
| pKB12 | pGP20, Δ*yscW*(Δ+113 to +298)^b^ *lcrF’-‘lacZ*, ori pSC101, Tet^R^ | this study |
| pKB14 | pBAD18-*lacZ*(481), pBAD::*lcrF-lacZ* (-124 to +74)^a^, ori pMB1, Ap^R^ | [[7](#_ENREF_34)] |
| pKB28 | pACYC184, P*_yscW_::yscWlcrF*(-1076 to +893)^a^, ori p15A, Tet^R^ | this study |
| pKB60 | pHSG576, *csrA*^+^, ori pSC101, Cm^R^ | this study |
| pKB63 | pTS02,*csrA-lacZ* (-1071 to +17)^c^ , ori pSC101, Ap^R^ | [[16](#_ENREF_79)] |
| pKB99 | pED07-*kan*, pBAD::*lcrF-lacZ* (-124 to +74)^a^, mutation GUU-30/-28AAA), ori pMB1, Kn^R^, Ap^R^ | this study |
| pKD4 | kanamycin cassette template, Kn^R^ | [[17](#_ENREF_80)] |
| pMP1 | pBAD33-RBS*_lcrF_*, 3’-UTR, ori M13, Cm^R^ | this study |
| pRS1 | pFU50, ori29807, Cm^R^ | this study |
| pRS2 | pRS1, *yopD*^+^, ori29807, Cm^R^ | this study |
| pRS4 | pRS1, *lcrQ*^+^, ori29807, Cm^R^ | this study |
| pRS15 | pRS1, ori p15A, Cm^R^ | this study |
| pRS16 | pRS2, *yopD^+^*, ori p15A, Cm^R^ | this study |
| pRS18 | pRS4, *lcrQ*^+^, ori p15A, Cm^R^ | this study |
| pRS30 | pDM4, Δ*yscS*, Cm^R^ | this study |
| pRS34 | pDM4, Δ*yopD*, Cm^R^ | this study |
| pRS40 | pBAD33, P_BAD_::*rne* (-15 to +1395)^d^, ori p15A, Cm^R^ | this study |
| pRS50 | pAKH3, Δ*pnp*, Ap^R^, Kan^R^ | this study |
| pSR1 | pIV2mob, *gfp_mut3.1_*, ori p29807, Kn^R^ | this study |
| pTS02 | pGP20, promoterless *lacZ*, ori pSC101, Ap^R^ | [[7](#_ENREF_34)] |
| pTS03 | pGP20, *lacZ*^+^, ori pSC101, Ap^R^ | [[7](#_ENREF_34)] |
| pTS31 | pFU98, *yadA-luxCDABE* +RBS, ori pSC101*, Cm^R^ | [[14](#_ENREF_27)] |
| pTS32 | pFU98, *invA-luxCDABE* + RBS, ori pSC101*, CmR | [[14](#_ENREF_27)] |
| pTS34 | pWO8, *lcrF-luxCDABE* + RBS, ori pSC101*, Cm^R^ | this study |
| pWO3 | pSR1 Δ*yscW*(Δ+113 to +298)^b^ *lcrF-gfp*, ori p29807, Kn^R^ | this study |
| pWO8 | pFU51, P*_yscW_::ycsWlcrF*, ori SC101*, Ap^R^ | this study |
| pWO13 | pWO8, P*_yscW_::ycsWlcrF,* ori R6K mob, Ap^R^ | this study |
| pWO14 | pFU51 P*_yscW_::ycsWlcrF*, insertion sequence, ori R6Kmob, AmpR | this study |
| pWO41 | pTS31 *yadA-luxCDABE* + RBS, SC101*, Amp^R^ | this study |
| pWO42 | pTS34 *lcrF-luxCDABE* +RBS, SC101*, Amp^R^ | this study |
| pZA31luc | Expression vector, p15A, P*_LtetO-1_*, Cm^R^ | [[18](#_ENREF_81)] |

^a^ – relative to translational start codon of *lcrF*

^b^ – relative to transcriptional start site of *yscW*

^c^ – relative to translational start codon of *csrA*

^d^ – relative to transcriptional start site of *rne*
