# Supplementary material for: A bacterial secreted translocator hijacks riboregulators to control type III secretion in response to host cell contact
Source: PLoS Pathog. 2019 Jun 7;15(6):e1007813. doi: 10.1371/journal.ppat.1007813 (PMC6583979; doi:10.1371/journal.ppat.1007813)
Supplement: S2 Table — The table includes all oligonucleatides used in this study. (DOCX) [file ppat.1007813.s002.docx]

**Table S2: Oligonucleotides**

| **Name** | **Sequence (5’-3’)** | **Restriction site(s)** |
| --- | --- | --- |
| 90 | CGCGGCGGATCCCCTTAACGTACGTTTTCGTCCC | *Bam*HI |
| 92 | CGCGGCGGTACCCGCAGATATTAATGCCGCAGAGAC | *Bam*HI |
| 555 | CGGCGCGGATCCCTCTCACACCAGCTGTG | *Bam*HI |
| 556 | GGGGGCGTCGACGGCAAACTCAATATCCTG | *Sal*I |
| 558 | CGGCGCGGATCCGGACAATGGTCGATGAC | *Bam*HI |
| 559 | GGGGGCGTCGACGTTACACGAGACGCTGC | *Sal*I |
| 583 | GGGCGCGGATCCGATTGGGCCGGAATCTAGC | *Bam*HI |
| I68 | CGGCGCCTCGAGGTAAGTCGTCGGTTGAGAC | *Xho*I |
| I82 | GCAATCAGCTAGTCAATTTG |  |
| I214 | GCGGCGTCTAGACCATTGAATCTTCACAATCTAATCCCG | *Xba*I |
| I222 | GCGGCTGCAGCCATCTTGTGAATGCTCAACAACC | *Pst*I |
| I224 | GCGGCCTGCAGGGCTGCAATGTAACTAGGAATATGG | *Pst*I |
| I303 | GCGCGCTGCAGGATTTTTAGGACAGTATAAC | *Pst*I |
| I404 | TTTGAATTCGCCATCTTGTGAATGCTCAAC | *EcoRI* |
| I515 | GCAGTTCATTTGGATCAATAC |  |
| I661 | GTGTAGGCTGGAGCTGCTTC |  |
| I662 | CATATGAATATCCTCCTTAGTTCC |  |
| I746 | CGTATGTCACGACCATTATCTCGCGTGGCTCAGCAAAAG |  |
| I747 | CTTTTGCTGAGCCACGCGAGATAATGGTCGTGACATACG |  |
| I844 | GCGGCGCATGCGGCTGCAATGTAACTAGGAATATGG | *Sph*I |
| I964 | GCACTGGATCCCTAATCTTGTGAATGCTCAACAACC | *Bam*HI |
| I972 | GCATAGATCTTACGGGGTCTGACGCTCAGTG | *Bgl*II |
| I998 | GCATCAGATCTTCTGTTGTTTGTCGGTGAACG | *Bgl*II |
| II275 | GGGGCCCTGCAGCGAGTCAGAATAAGCATTCTTTG | *Pst*I |
| II341 | GCACGGAGCTCAGGCGAGTATCGTGATAG | *Sac*I |
| II342 | GCACGGAGCTCGTCAGGTGGTCAACCTTCT | *Sac*I |
| II360 | CGGCTGCAGGCGACAGGAGACTCGATG | *Pst*I |
| II361 | CGGGCGGCCGCCAGTAGATATATATTATCTCAGC | *Not*I |
| II363 | CGGACTAGTCAGGCAACACAAGTCGCCG | *Spe*I |
| II364 | CGGGCATGCGCAAAATGCCGATCACAGCC | *Sph*I |
| II365 | GGAATAACCATGACAATAAATATCGGTGTTGTCTGACCATTGATG |  |
| II366 | CATCAATGGTCAGACAACACCGATATTTATTGTCATGGT TAT TCC |  |
| II496 | GCGCGTTATAAGCTGTCAAACATGAGAATTACAAC | *Psi*I |
| II538 | GCGCGGAGCTCCCGCCGGACATCAGCGC | *Sac*I |
| III233 | GCGCGCTCTAGAAGGAGGTTGTTAAAGATGAAAAGAATGTTGATTAACGCG | *Xba*I |
| III234 | GCGCGCGCATGCTTATTAACGCTTCTCAATAGCGTTTACTG | *Sph*I |
| III243 | GCGGCGGAGCTCGTTGCTGACTTCGGTCGCGG | *Sac*I |
| III244 | **GAAGCAGCTCCAGCCTACA**CGCAAAATTGTACCCTTATGCTTTGC |  |
| III245 | **ACTAAGGAGGATATTCATATG**GTAATTAAGTCATTCCACAGCTCC |  |
| III246 | GCGGCGGAGCTCGCTCTAGTACAGAATCAAACG | *Sac*I |
| III394 | CCTCGTTCATAAGCACTCGTC |  |
| III645 | GGGCGCACTAGTTCAGACAACACCAAAAGCG | *Spe*I |
| III647 | GGGCGCCTCGAGTCATCATGGGTTATCAACGCAC | *Xho*I |
| III731 | GAATAAGCATTCTTTGCTCC |  |
| III902 | GGGCGCGCATGCGCTGACTCATACCAGGCC | *Sph*I |
| III905 | GGGCGCTCTAGAGCTCTGCCAGTGTTACAAC | *Xba*I |
| V066 | GGGCGCGTAATACGACTCACTATAGGAATGTAATGGCTTACGTTTTC |  |
| V708 | GGGCGCGTAATACGACTCACTATAGAACAGAGAGACCCGACTC |  |
| VIII671 | **AAACCCCTCCGTTTAGAGAGGGGTTATGCTAGTT**Accggttattattatttttgacacc |  |
| IV436 | GGGGCCGAATTCGCCAGCGGCATTAG | *Eco*RI |
| IV438 | GGGGCCGAATTCGGACTCGACCAAGCTACTTAC | *Eco*RI |
| IV527 | TTGCTGACTCCGATTATTCG |  |
| IV528 | GAAGACGACCGCGCCCAAC |  |
| IV529 | CGCGACTCAGCAAGAAGAG |  |
| IV530 | GCCGATGTCTGGGCGCAG |  |
| IV706 | GGGCGCCATATGATGACAATAAATATCAAGACAG | *Nde*I |
| IV708 | GGGCGCACTAGTGTGATGCAACGTCTGCTAGATG | *Spe*I |
| IV783 | GCGCCCCATGGATGCTTATTCTGACTCG | *Nco*I |
| V066 | GGGCGCGTAATACGACTCACTATAGGAATGTAATGGCTTACGTTTTC |  |
| V631 | GTTTCCCCGCCATTATCC |  |
| V659 | GCGGCGGAGCTCGTGATTTATTATATTGGTTTTGGTTG | *Sac*I |
| V700 | GGGCGCGTAATACGACTCACTATAGCAACAATACCGTGAAATGC |  |
| V708 | GGGCGCGTAATACGACTCACTATAGAACAGAGAGACCCGACTC |  |
| V731 | GGGCGCGTAATACGACTCACTATAGGGTGATTTATTATATTGGTTTTGGTTG |  |
| V732 | CTCTAGTGATGCCATAAATGTTATAC |  |
| V830 | GGGCGCGTAATACGACTCACTATAGCTTACATTTTATATGAATGTAATGGC |  |
| V831 | CATACACCGTGAAAACGTAAG |  |
| V832 | GGGCGCGTAATACGACTCACTATAGCACGGTGTATGATGGATAATG |  |
| VI085 | CGGAGTCAGCAAAATTGTACC |  |
| VI386 | GCGCGTAATACGACTCACTATAGGGCGGGGAAACAGAGAG |  |
| VI564 | GGCTAATACGACTCACTATAGGGCTTACCGAAATAATGCG |  |
| VI565 | CATTCTTTTCATCTTTAACTTACTCG |  |
| VI950 | GCGCGTAATACGACTCACTATAGGTTCGCGCGGCTAATGAGAG |  |
| VIII672 | GCGCCGGATCCCGAACGTTTAGGTCTGCGTC | *Bam*HI |
| VIII673 | GCGCCGTCGACCTAATGACAATCCTTAACTTTC | *Sal*I |
| VIII674 | GCGCCGTCGACGTATGTTGACCATACTGGAAT | *Sal*I |
| VIII675 | GCGCCGGATCCGTTATCGATCCGCTGACCG | *Bam*HI |
| VIII676 | GCGCCGTCGACGTTTATTATTCCACAAGTCTCTTGG | *Sal*I |
| VIII677 | GCGCCGTCGACCGCAACTCTTCTTGCTGAG | *Sal*I |
| **qRT-PCR Primer** |  |  |
| III393 | CCGACGTAAAGCCGCGATAC | *sopB* (fw) |
| III394 | CCTCGTTCATAAGCACTCGTC | *sopB* (rev) |
| IV529 | CGCGACTCAGCAAGAAGAG | *rne* (fw) |
| IV530 | GCCGATGTCTGGGCGCAG | *rne* (rev) |
| IV931 | CGCCCAGACGCGCTTCGGCC | *pnp* (rev) |
| IV946 | GTTGGGCGCGGTCGTCTTCGG | *pnp* (fw) |
| V89 | CAGGGACTGCTTAGGACGAG | *csrB* (fw) |
| V90 | CCTGCTCAATCCCTGAAAAC | *csrB* (rev) |
| VI98 | GGAGCACAGTTACTCAGGATGAG | *csrC* (fw) |
| VI99 | GGCGATTCGCCCGGCGCTC | *csrC* (rev) |

Underlined – restriction sites

Dotted underlined – T7 promoter

Bold – sequences homologous to kanamycin resistance gene (pKD4)

Bold and underlined – terminator sequence
