# Supplementary material for: A bacterial secreted translocator hijacks riboregulators to control type III secretion in response to host cell contact
Source: PLoS Pathog. 2019 Jun 7;15(6):e1007813. doi: 10.1371/journal.ppat.1007813 (PMC6583979; doi:10.1371/journal.ppat.1007813)
Supplement: S1 References — (DOCX) [file ppat.1007813.s003.docx]

**Table S1 References:**

1. Manoil C, Beckwith J. A genetic approach to analyzing membrane protein topology. Science. 1986;233:1403-8.

2. Simon R, Priefer U, Puehler A. A broad host range mobilization system for *in vivo* genetic engineering: transposon mutagenesis in gram negative bacteria. Biotechnology. 1983;1:784-91.

3. Studier FW, Moffatt BA. Use of bacteriophage T7 RNA polymerase to direct selective high-level expression. J Mol Biol. 1986;189:113-30.

4. Bolin I, Norlander I, Wolf-Watz H. Temperature-inducible outer membrane protein of *Yersinia pseudotuberculosis* and *Yersinia enterocolitica* is associated with the virulence plasmid. Infect Immun. 1982;37:506-12.

5. Dersch P, Isberg RR. A region of the *Yersinia pseudotuberculosis* invasin protein enhances integrin-mediated uptake into mammalian cells and promotes self-association. EMBO J. 1999;18(5):1199-213. Epub 1999/03/04. doi: 10.1093/emboj/18.5.1199. PubMed PMID: 10064587; PubMed Central PMCID: PMC1171211.

6. Heroven A, Bohme K, Rohde M, Dersch P. A Csr-type regulatory system, including small non-coding RNAs, regulates the global virulence regulator RovA of *Yersinia pseudotuberculosis* through RovM. Mol Microbiol. 2008;68(5):1179-95. Epub 2008/04/24. doi: MMI6218 [pii] 10.1111/j.1365-2958.2008.06218.x. PubMed PMID: 18430141.

7. Böhme K, Steinmann R, Kortmann J, Seekircher S, Heroven AK, Berger E, et al. Concerted actions of a thermo-labile regulator and a unique intergenic RNA thermosensor control *Yersinia* virulence. PLoS Pathog. 2012;8(2):e1002518. Epub 2012/02/24. doi: 10.1371/journal.ppat.1002518. PubMed PMID: 22359501; PubMed Central PMCID: PMC3280987.

8. Chang AC, Cohen SN. Construction and characterization of amplifiable multicopy DNA cloning vehicles derived from the P15A cryptic miniplasmid. J Bacteriol. 1978;134(3):1141-56.

9. Bücker R, Heroven AK, Becker J, Dersch P, Wittmann C. The pyruvate-tricarboxylic acid cycle node: a focal point of virulence control in the enteric pathogen *Yersinia pseudotuberculosis*. J Biol Chem. 2014;289(43):30114-32. doi: 10.1074/jbc.M114.581348. PubMed PMID: 25164818; PubMed Central PMCID: PMC4208018.

10. Waldminghaus T, Fippinger A, Alfsmann J, Narberhaus F. RNA thermometers are common in alpha- and gamma-proteobacteria. Biol Chem. 2005;386(12):1279-86. Epub 2005/12/13. doi: 10.1515/BC.2005.145. PubMed PMID: 16336122.

11. Guzman LM, Belin D, Carson MJ, Beckwith J. Tight regulation, modulation, and high-level expression by vectors containing the arabinose P*_BAD_* promoter. J Bacteriol. 1995;177(14):4121-30.

12. Milton DL, O'Toole R, Horstedt P, Wolf-Watz H. Flagellin A is essential for the virulence of *Vibrio anguillarum*. J Bacteriol. 1996;178(5):1310-9.

13. Strauch E, Voigt I, Broll H, Appel B. Use of a plasmid of a *Yersinia enterocolitica* biogroup 1A strain for the construction of cloning vectors. J Biotechnol. 2000;79(1):63-72.

14. Uliczka F, Pisano F, Kochut A, Opitz W, Herbst K, Stolz T, et al. Monitoring of gene expression in bacteria during infections using an adaptable set of bioluminescent, fluorescent and colorigenic fusion vectors. PLoS One. 2011;6(6):e20425. doi: 10.1371/journal.pone.0020425. PubMed PMID: 21673990; PubMed Central PMCID: PMC3108616.

15. Takeshita S, Sato M, Tabo M, Masahashi W, Hashimoto-Gothoh T. High-copy-number and low-copy-number plasmid vectors for *lacZ* a-complementation and chloramphenicol- or kanamycin resistance selection. Gene. 1987;61:63-74.

16. Nuss AM, Schuster F, Kathrin Heroven A, Heine W, Pisano F, Dersch P. A direct link between the global regulator PhoP and the Csr regulon in *Y. pseudotuberculosis* through the small regulatory RNA CsrC. RNA Biol. 2014;11(5):580-93. PubMed PMID: 24786463.

17. Datsenko KA, Wanner BL. One-step inactivation of chromosomal genes in *Escherichia coli* K-12 using PCR products. Proc Natl Acad Sci U S A. 2000;97(12):6640-5. PubMed PMID: 10829079.

18. Lutz R, Bujard H. Independent and tight regulation of transcriptional units in *Escherichia coli* via the LacR/O, the TetR/O and AraC/I1-I2 regulatory elements. Nucleic Acids Res. 1997;25(6):1203-10. PubMed PMID: 9092630.
