# Supplementary figures and images for: A bacterial secreted translocator hijacks riboregulators to control type III secretion in response to host cell contact
Source: PLoS Pathog. 2019 Jun 7;15(6):e1007813. doi: 10.1371/journal.ppat.1007813 (PMC6583979; doi:10.1371/journal.ppat.1007813)

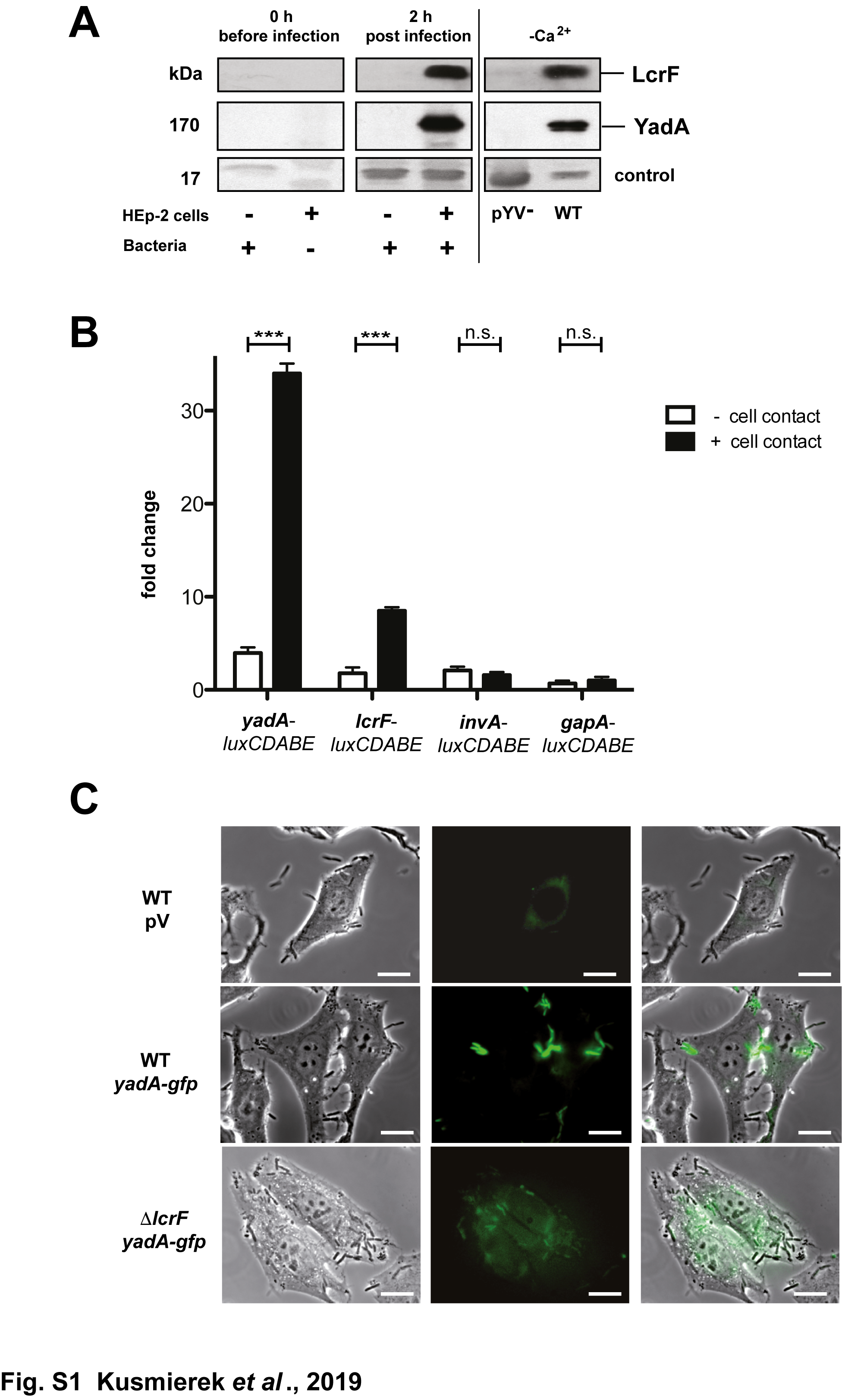

Supplement: S1 Fig — (A) Left panel: extracts of strain YPIII (WT) resuspended in the cell culture supernatant (SN) and extracts of uninfected HEp-2 cells. Middle panel: strain YPIII (WT) incubated for 2 h at 25°C with (right) or without (left) seeded HEp-2 cells. Right panel: strain YPIII (WT) and the isogenic virulence plasmid-cured strain (pYV-) grown in LB under secretion conditions (37°C, in the absence of Ca2+). After 2 h HEp-2 cells were lysed with 0.1% Triton, whole cell extracts were prepared and YadA and LcrF were detected by western blotting using polyclonal antibodies directed against YadA and LcrF. Polyclonal anti-H-NS antibodies were used to detect H-NS, which served as loading control. Furthermore, whole cell extracts of the virulence plasmid-cured strain YP12 (pYV-) and the wildtype strain YPIII (WT) grown under secretion conditions at 37°C in the absence of Ca2+ (right panel) were used as negative and positive controls. (B) YPIII (WT) strains harboring lcrF-luxCDABE (pTS34), yadA-luxCDABE (pTS31), invA-luxCDABE (pTS32), or gapA-luxCDABE (pFU166) fusion plasmids, were used to infect HEp-2 cells in PBS or incubated in PBS without cells at 25°C. Bioluminescence of the samples was monitored after 2 h. The data represent the mean ± SD of the fold change (end/start) from three independent biological replicates and were analyzed with Student’s t-test: ***: P<0,001, n.s. (P>0.05). (C) HEp-2 cells infected with Y. pseudotuberculosis wildtype strain YPIII harboring pJE2 (empty vector) or a plasmid-encoded yadA-gfp (pJE9) and ΔlcrF strain carrying plasmid-encoded yadA-gfp (pJE9) incubated for 4 h at 25°C. White bars indicate 5 μm. (TIF) [file ppat.1007813.s004.tif]

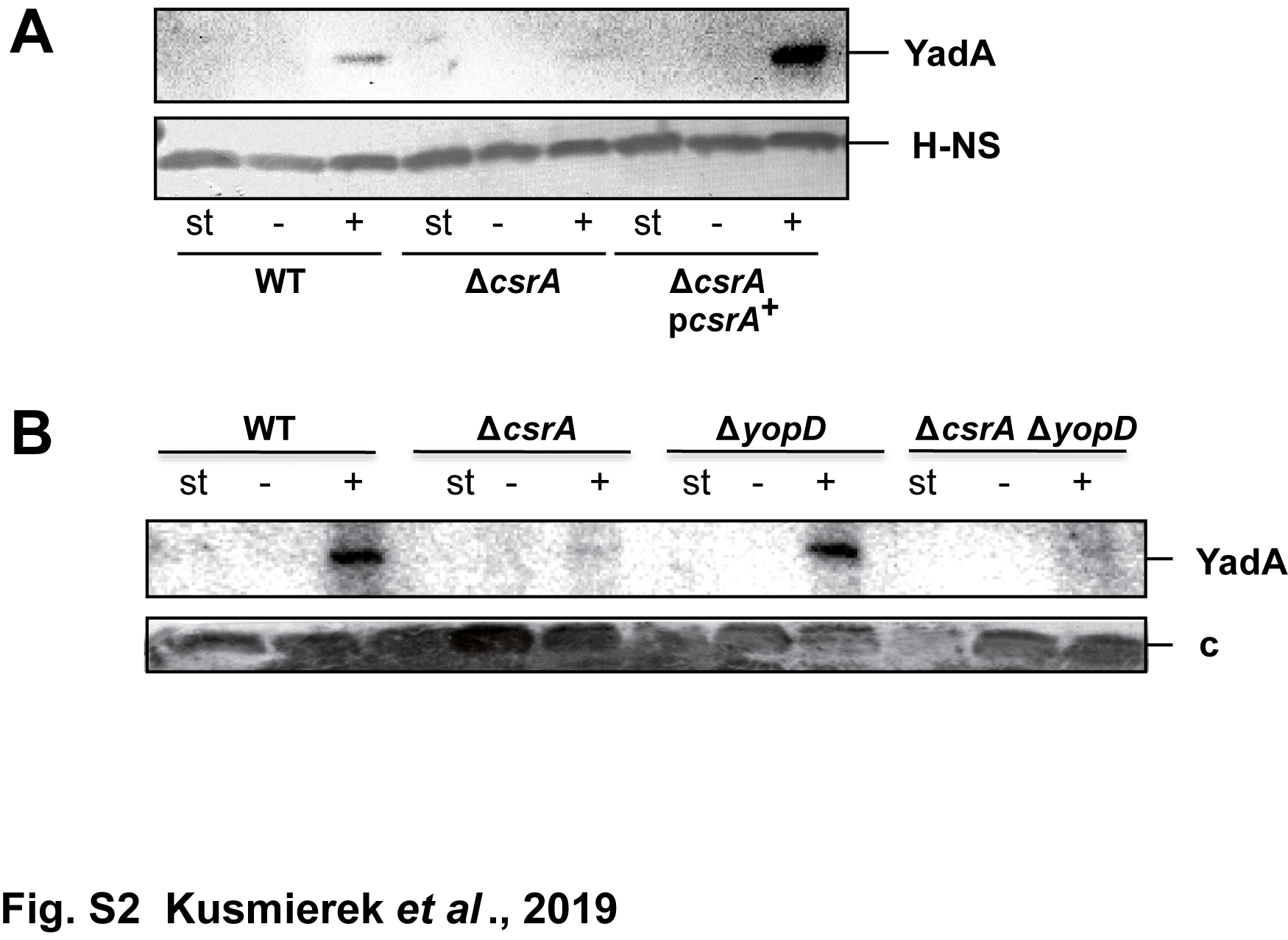

Supplement: S2 Fig — (A) HEp-2 cells were infected with YPIII (WT), YP53 (ΔcsrA) and YP53 pAKH56 (ΔcsrA) pcsrA+ at 25°C (+). After 2 h the cells were lysed with 0.1% Triton and samples for SDS-polyacrylamide electrophoresis and western blot analysis with polyclonal anti-YadA or anti-H-NS (control) sera were prepared. YadA expression was visualized in bacteria incubated in PBS without cells (-) or with HEp-2 cells (+) or in bacteria that were used to start HEp-2 infection (st). (B) HEp-2 cells were infected with YPIII (WT), YP53 (ΔcsrA), YP91 (ΔyopD), and YP145 (ΔcsrA, yopD) at 25°C (+). After 2 h samples for SDS-polyacrylamide electrophoresis and western blot analysis with polyclonal anti-YadA and anti-H-NS (control) sera were prepared. YadA expression was visualized in bacteria incubated in PBS without cells (-) or with HEp-2 cells (+) or in bacteria that were used to start HEp-2 infection (st). (TIF) [file ppat.1007813.s005.tif]

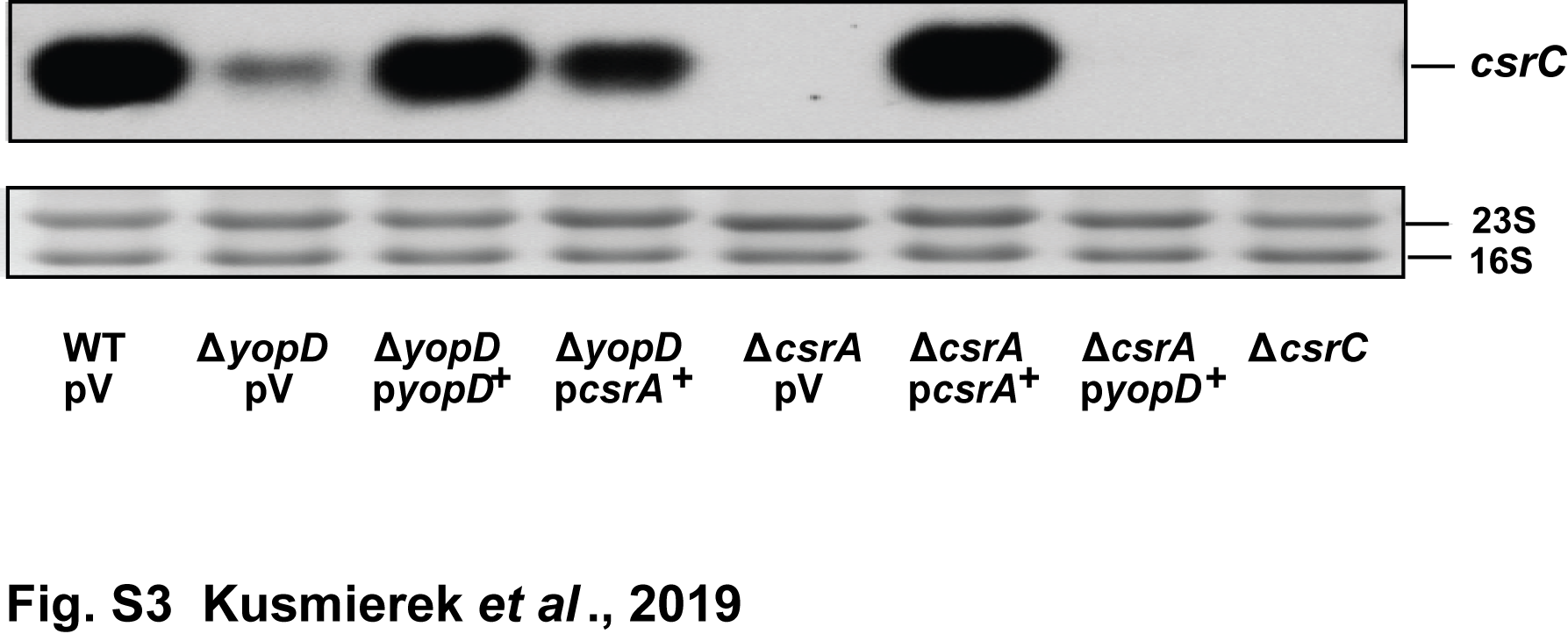

Supplement: S3 Fig — Y. pseudotuberculosis strain YPIII (WT) pV (empty vector pRS1), YP91 (ΔyopD) pV (empty vector pRS1), YP91 (ΔyopD) pyopD+ (pRS2), YP53 (ΔcsrA) pV (empty vector pRS1), YP53 (ΔcsrA) pcsrA+ (pKB60), YP53 (ΔcsrA) pyopD+ (pRS2) were used to analyze reciprocal complementation of YopD and CsrA of a csrA and yopD mutant by monitoring CsrC transcript levels. The csrB-deficient strain YP52 was used as negative control. Total RNA of the cultures was prepared and subjected to northern blotting using a CsrC-specific probe. The 16S and 23S rRNAs served as loading controls. A representative of at least three independent experiments is presented. (TIF) [file ppat.1007813.s006.tif]

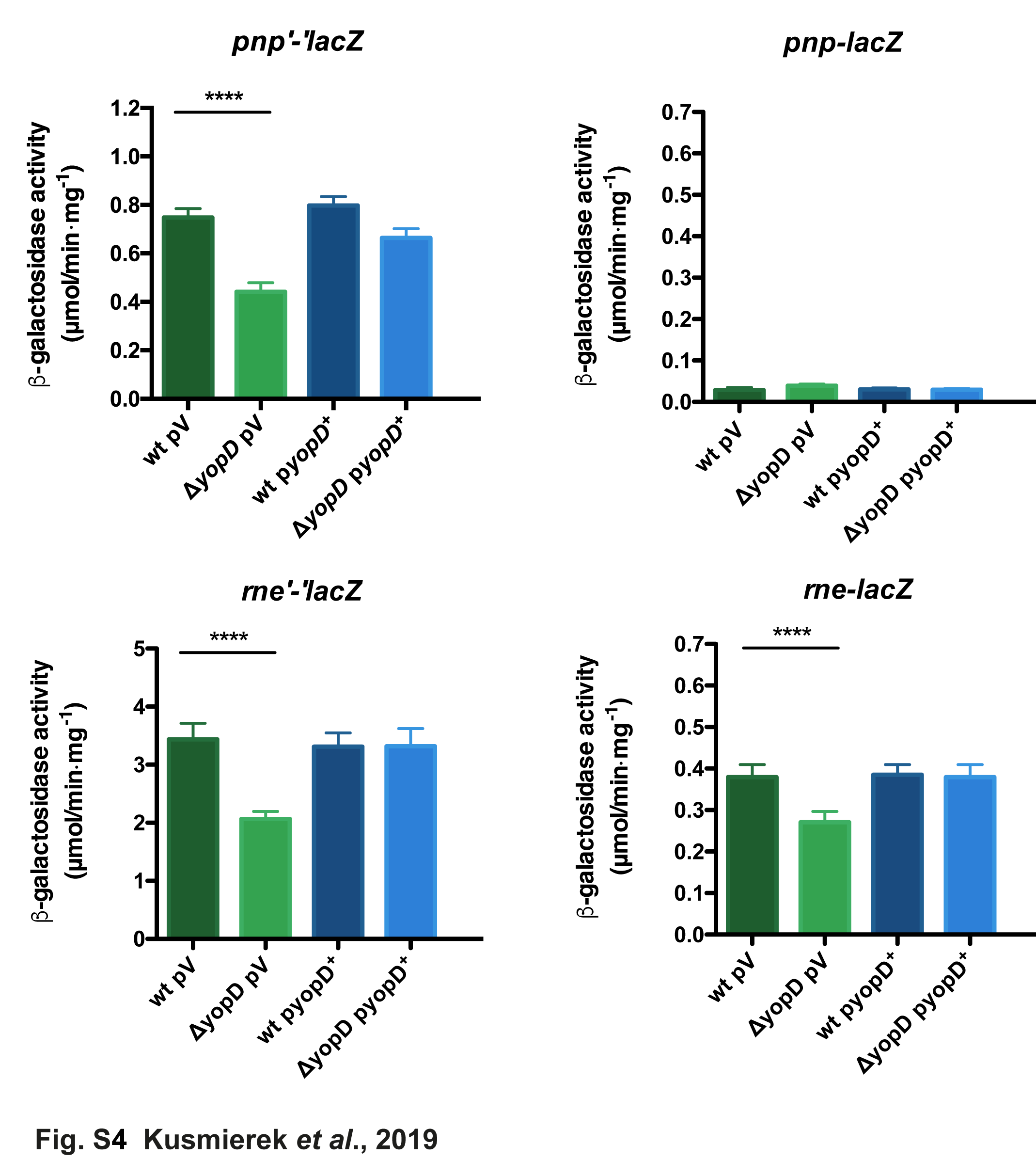

Supplement: S4 Fig — Strains YPIII (WT) pRS15 (empty vector), the YP91 (ΔyopD) pRS15 (empty vector), and YP91 (ΔyopD) pRS16 (yopD+) harboring transcriptional or translational rne-lacZ or pnp-lacZ reporter plasmids were grown to late exponential phase at 37°C. β-galactosidase activity of the different cultures was monitored. The data represent the mean ± SD from at least three independent biological replicates performed in triplicates and were analyzed with Student’s t-test; ****: P<0,0001. (TIF) [file ppat.1007813.s007.tif]
